# Untargeted metabolomics as a new perspective for assessing taxonomic boundaries in alpine *Rubus* genus

**DOI:** 10.1371/journal.pone.0354840

**Published:** 2026-08-05

**Authors:** Emiliano Serviole, Antoine Henriot, Christiane Gallet, Coralie Audoin

**Affiliations:** 1 Grenoble Alpes University, Savoie Mont Blanc University, CNRS, Laboratoire d’Ecologie Alpine, Grenoble, France; 2 Laboratoires Clarins, Pontoise, France; 3 Asters, Conservatoire d’espaces naturels de Haute-Savoie, Annecy, France; Foshan University, CHINA

## Abstract

The development of a robust taxonomy for the genus *Rubus* remains a real challenge, due to apomictic reproduction and hybridization that led to a very large number of “taxa” with undefined frontiers. Using an untargeted metabolomic approach and a two-year leaf sampling along an elevational gradient in the French Alps we provide new tools to assess boundaries between nine *Rubus* taxa. Multivariate analyses on ionic intensity matrices (of 566 and 668 features for 2023 and 2024 sampling respectively) have shown that *R. idaeus* species, the two sections *Corylifolii* and *Rubus*, and *Caesii* section related taxa, formed three distinct super groups. Thirty-eight compounds have been annotated, including 8 detected in genus *Rubus* for the first time. Discriminant compounds have been annotated, allowing to propose ursane triterpenoids, flavonoids glycosides, lignans and gallotannins as taxonomic classifiers of the genus *Rubus*. Among those, quercetin – and kaempferol – 3-O-methylglutaryl hexoside were detected at higher levels in *R. idaeus*, whereas neolignans glycosides have been mostly detected in sections *Corylifolii* and *Rubus*. The intermediate position of *Caesii* between the two others did not allow to propose specific biomarkers of the section. The need for a precise and detailed description of the phytochemical profiles of the various *Rubus* subgroups is reinforced by the growing use of some of these compounds in pharmacology and cosmetics.

## Introduction

*Rubus* L. (brambles), part of the *Rosaceae* family has one of the widest distribution range among the flowering plants and is particularly well represented in Europe [1–3]. Some of its representatives have been largely exploited for their edible fruits such as raspberries and blackberries (*R. idaeus* and *R. fruticosus aggr.* y.). The genus *Rubus* is a complex group in terms of taxonomy. The number of species to be considered within this genus varies according to the authors and the areas studied but remains in all cases substantial and highly controversial.

The lack of consensus regarding the taxonomy of brambles is mainly due to apomixis, which affects speciation and is the cause of the multiplication of local taxa [4–7]. Apomixis is an asexual reproduction mode producing viable seeds from maternal ovule tissue without fertilization [8], with multiple types (strict or facultative) involving diverse cellular mechanisms [9]. Retention of sexuality in some of these species [10] can promote hybridization phenomena that complicate the classification of the genus to which they belong [6] by producing a large number (often hundreds) of taxa, morphologically similar and that behave biologically like species without consensus on their taxonomic classification [6,11]. The instability of the taxonomic ranks used in classification [11], and the presence of regional or even local taxa [6], result in either an underestimation of the real number of taxa by using aggregates rank, or on the contrary an overestimation by using the rank of subspecies.

**Fig 1:**
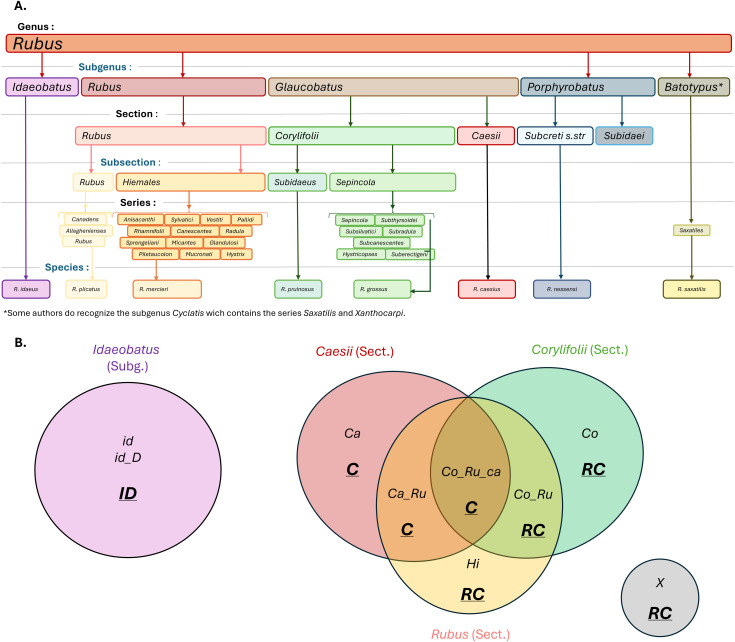
*Rubus* taxonomic classification and sampling groups. **A**: Adapted taxonomic classification of European *Rubus* taxa from works of [4,5,16–20]. **B**: Repartition of 8 morphological groups in 3 super groups (underlined) defined by metabolomics analysis. *X* represents sample group with no morphological assignment.

The genus *Rubus* is considered to be one of the most difficult in the European flora in terms of taxonomic classification, which has led to the production of limited and confusing literature [1,3,12–15]. The multitude of taxa has encouraged the batologists (botanists specialized in brambles) to reduce the number of taxa to be considered [1,5,6]. Although the complex state of bramble taxonomy, the following hierarchical classification is the most widely used nowadays: Genus; subgenus; section; sub-section; series; species (Fig 1A). This study concerns the five subgenera generally described in European *Rubus*, and the diverse and numerous taxa they include (Fig 1A). The organization within this reduced range of *Rubus* taxa presents some taxonomical divergences and complexities, such as the frequent hybridization events between *R. caesius* (Sect. *Caesii*) and other sections: mainly Sect. *Rubus* [3,5,12,21] but also with the species *R. idaeus* [4,14], creating overlapping morphological criteria of identification and unstable classification concerning those taxa. Moreover, some authors consider other subgenus, such as *Anoplobatus*, *Chamerubus*, *Cyclactis* that sometimes encompass species belonging to the sections mentioned before [22,23] and tend to blur the taxonomical boundaries.

Phenolic compounds, including tannins, various flavonoids and phenolic acids have been largely described in different parts of the plants in all *Rubus* taxa investigated [24]. Terpenoids have also been described, including labdane-type diterpenes ursane- and oleanane-type triterpene glucosides, suaviosides and triterpenoids [25–27]. Those compounds probably explained the traditional and worldwide traces of therapeutic uses of *Rubus* leaves, as infusion to treat numerous ailments [28,29]. Still, no attempt to use such compounds for chimiotaxonomic purposes have been found.

Recently, some studies have shown that metabolomics could provide relevant data to better understand and clarify taxonomic issues within genus such as *Myrothamnus*, *Viola* and *Ocotea* [30–33]. We therefore question the links between the taxonomic complexity and the diversity of chemical profiles in *Rubus* taxa, using leaf samples from alpine environment that maximize the metabolic variability [34–37] analyzed by untargeted metabolomics (LC-MS-ESI-QTOF). Using a two-year sampling of bramble leaves along an elevational gradient in the French Alps, we ask whether morphologically identical taxa share similar chemical profiles and whether specific metabolites can serve as taxonomic markers within *Rubus*.

## Results and discussion

### Multivariate analysis

Ethanol extracts were prepared from dried leaves and UPLC-MS based metabolomic analysis was performed for all samples for both years separately, in positive electrospray ionization (ESI). A similar analysis in negative electrospray ionization was conducted separately on QC samples, and one sample from each taxonomic group (according to morphological identification) from 2024’s sampling, to obtain supplementary spectral informations for compounds which may have a better negative ionization, such as hydrolysable tannins and (glycosylated) flavonoids. Only the data obtained in positive electrospray ionization were processed in Mzmine to obtain ionic intensities matrices for each year (2023: 53 x 566; 2024: 192 x 668).

**Fig 2:**
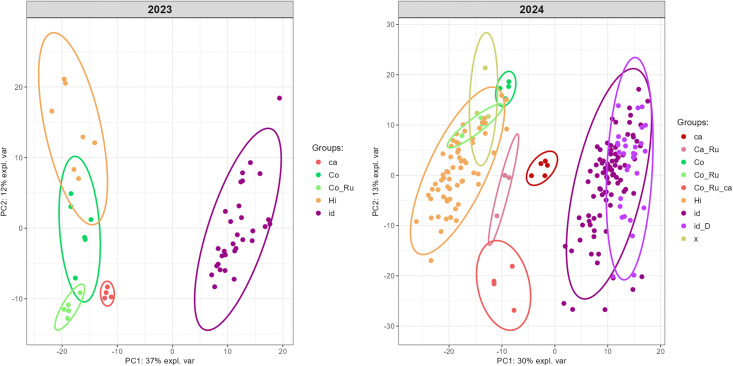
Principal Component Analysis of the ionic intensity matrices from the LC-MS analysis of *Rubus* leaves: sampled in Left: 2023 (53 samples x 566 features) and Right: 2024 (192 samples x 668 features).

**Fig 3:**
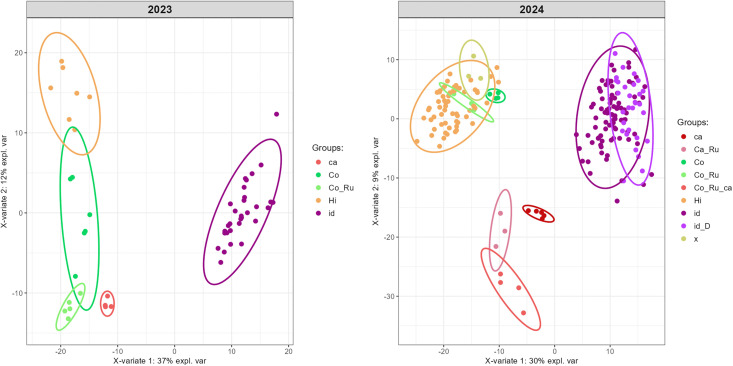
Partial Least Squares Discriminant Analysis of the ionic intensity matrices from the LC-MS analysis of *Rubus* leaves: sampled in Left: 2023 (53 samples x 566 features) and Right: 2024 (192 samples x 668 features).

**Fig 4:**
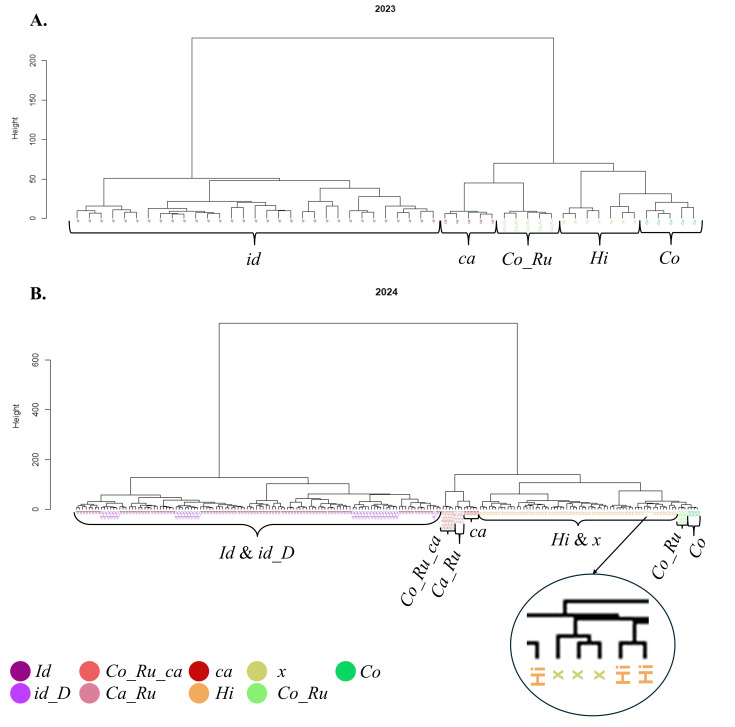
Dendrograms from the hierarchical clustering analysis illustrating the clustering of the samples from the LC-MS analysis of *Rubus* leaves: sampled in A: 2023 and B: 2024.

The principal component analysis (PCA) (Fig 2), the Partial Least Squares – Discriminant Analysis (PLS-DA) (Fig 3), and the hierarchical cluster analysis (HCA) (Fig 4) on both 2023 and 2024 matrices showed segregation into three groups which have been respectively assigned to 3 super groups: *ID*, *C*, and *RC* (Fig 1B). Those groups respectively correspond to the *Idaeobatus* subgenus (*ID*), the *Caesii* section (*C*), and the two sections *Rubus* and *Corylifolii* (*RC*).

The *ID* super group consisted of *R. idaeus* species which were classed in *id* (2023 and 2024) and *id_D* (2024) group according to morphological identification. Samples from super group *ID* were clearly segregated from other samples in all analyses for both years. Projection in PCA and PLS-DA showed a clear separation of the super groups along the first axes for each year (Figs 2, 3) and HCA’s first branch division splits *ID* supergroup from other samples (Fig 4) displaying a strong clustering of *R. idaeus* species according to Euclidean distances measures based on the ionic intensity of each sample. Both years show the same tendencies with the *R*. *idaeus* (*ID* supergroup) striking separation against the other taxa. Even if this species can be easily morphologically distinguished, our results support the taxonomic and evolutive distance of *R. idaeus* from other *Rubus* taxa already studied [19,38] and confirms their metabolomic divergences.

*C* super group consists of the morphological group *ca* (*R. caesius* species) (2023 and 2024) and the *Ca_Ru* and *Co_Ru_ca* groups (2024) that couldn’t be properly identified with morphological analysis, but both show morphological characteristics affiliated to the *Caesii* section and more precisely to *R. caesius* species. The third super group *RC* included the morphological group from the subsection *Hiemales* (*Hi*) part of *Rubus* section (Fig 1), and *Corylifolii* section (*Co*, *Co_Ru*) as well as the *x* group that couldn’t be identified. PCA and PLS-DA 2024’s projections showed a clear segregation of *C* super group along first axis, samples being located in the center between *ID* and *RC* (*Hi*, *Co*, *Co_Ru* and *x*). A notable difference between the two years is that in 2023 *Co_Ru* groups seemed to be more separated from the *Hi* group than from the *ca* group (Figs 2, 3). Multivariate analysis showed that *C* super group was segregated from *RC* and *ID* with a relevant clustering displayed by HCA analysis (Fig 4). Trees from both years (Fig 4A and 4B) showed a clear clustering of *ID* and *C* super groups. The remaining part of the trees contained all the samples from the *Hi*, *Co* and *Co_Ru* groups. In the 2023 tree, *Hi* group split among the *Co* group (Fig 4A) whereas in Fig 4B the *Co* and *Co_Ru* groups were well clustered together aside from the *Hi* samples. Finally, the *x* group from 2024 data was well clustered among the samples belonging to the *Hi* group (Fig 4B). The separation between the *RC* and *C* groups supports the taxonomical choice to separate the *Caesii* (*C*) and *Corylifolii* (part of *RC* group) sections. Our results also showed that, despite some slight differences, the *Rubus* and *Corylifolii* sections were hardly distinguishable in terms of morphological analysis as well as metabolomics analysis which is consistent with the frequent hybridization events reported in literature [5,12,21,19,39]. The variability observed within the super group *RC* between the two sampling years could be attributed to the complexity of morphological identification.

Multivariate analyses suggested robust trends, considering the cumulative explication percentage of both axes were 49, 43, 49 and 32% for PCA and PLS-DA projections from Figs 2A, 2B, 3A and 3B respectively. Moreover, the similarities of observed tendencies between two years data reflect a meaningful repeatability of the experiment. All results showed a consistency between the taxonomy of the genus *Rubus* based on morphological analysis and the chemical profile of the specimens. Indeed the *R. idaeus* species (*id* group), the *R. caesius* species (*ca* group) and related (*Ca_Ru* and *Co_Ru_ca* groups) were clearly separated from the *RC* group (*Rubus* and *Corylifolii* sections). Among the *RC* group, we observed a trend on which individuals belonging to the *Coryfolii* section and related (*Co* and *Co_Ru* groups) differed from the individuals of the Subsection *Hiemales* (*Hi* group). The observed trends in multivariate analysis results were consistent with the taxonomic ranking of *Rubus* genus classification (Fig 1).

Globally, multivariate analysis suggests strong phytochemical profile identities of taxonomic ranks within the genus *Rubus*, considering that samples collected along a large environmental gradient were clustered according to their morphological identification rather than by their sampling station.

### Compounds annotation

Considering the similarities of trends between 2023 and 2024 and the greater number of stations and samples in the 2024 dataset, this latter was chosen for annotation.

### Molecular network

**Fig 5:**
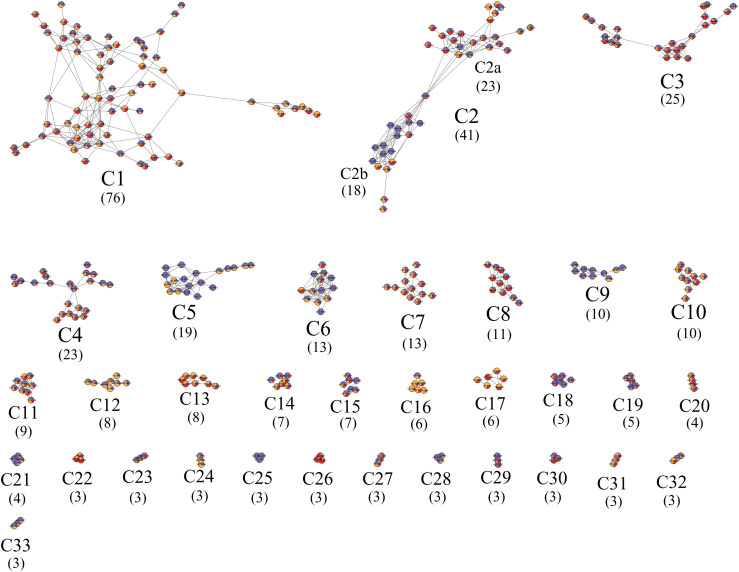
Molecular network based on MS/MS fragmentation by LC-MS ESI Q-TOF performed on *Rubus* leaf extracts. Color tag is based on super groups: Purple = *ID*, Red = *C*, Orange = *RC*; chart’s proportions were calculated by ionic intensity average of each feature in the three groups.

To have a global visualization of the features structural similarities based on their MS/MS fragmentation pattern, we built on Cytoscape a molecular network (Fig 5) of 668 nodes whom 349 were included in 33 clusters and 319 were duets and single nodes. The latter were not included in Fig 5 but the whole molecular network is available in supplementary (S1 Fig.).

### Distribution of the three super groups in the molecular network

Average ionic intensity (AII) of the features in the *ID*, *RC* and *C* super groups has been added in each node. Except for C7, C14, C27, C32, AIIs’ tendencies in favor of one or two groups have been observed in every cluster. Most nodes of cluster C1 exhibit higher AIIs for *C* and *RC* groups, as in the upper zone of the cluster C2 (C2a). In contrast, most of the nodes of the bottom part (C2b) exhibit higher AII for the *ID* group. Cluster C3 can also be divided into two parts: on the left nodes have an AII higher in the *ID* group, whereas in the right part nodes have an AII higher in the *RC* group. The following clusters are all characterized by a higher representation of the *ID* group: C4, C5, C6, C9, C15, C18, C21, C25, C28 and C33. Clusters C12, C16 and C24 were specific of *RC*, whereas Clusters: C22, C26 and C30 were specific of *C*. Finally, C10, C11, C13, C17, C20 and C31 have shown higher AIIs in *RC* and *C* groups, and ID and C specific clusters were C8, C19, C23 and C29.

### Annotation & propagation

**Fig 6:**
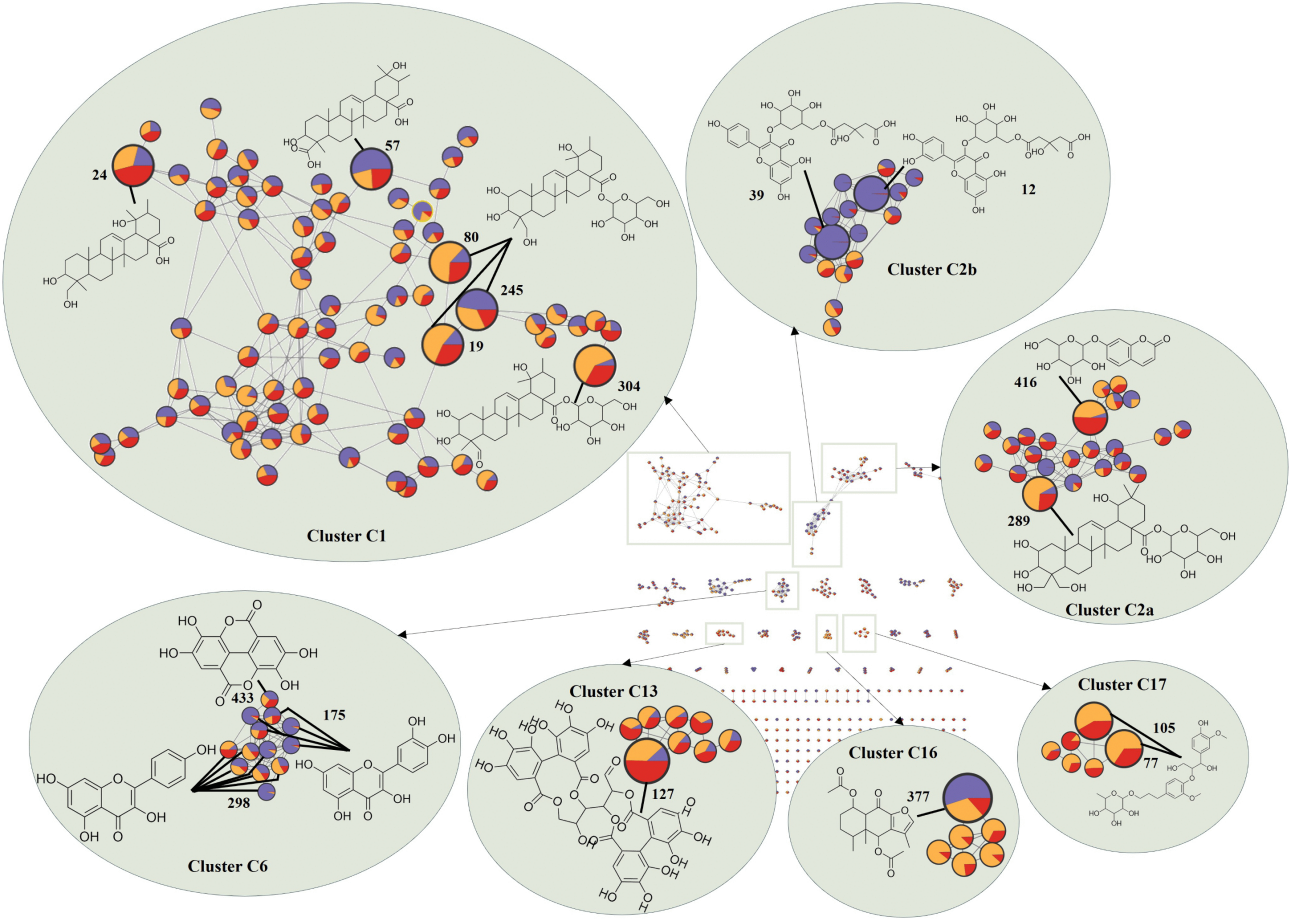
Molecular network based on MS/MS fragmentation by LC-MS ESI Q-TOF performed on *Rubus* leaf extracts and putative annotations (enlarged nodes with dash). Compound numbers refer to Table 1. Color tag is based on super groups: Purple = *ID*; Red = *C*; Orange = *RC*. Putative structures were based on LS-MS and MS/MS spectra, literature and databases matches.

Thirty-eight compounds were putatively annotated using spectral databases FragHub, homemade experimental library, GNPS, Fiora-fragmented Lotus and data literature (Table 1), and included mainly triterpenoids, lignans, flavonoids, and gallotannins. Compounds listed in Table 1 are level 2 annotations, while level 3 annotations were proposed using propagation based on molecular network clustering and MS/MS fragmentation [80]. Detailed mass spectrometry information for identification was provided as Supporting Information (S2-S3 Figs.). Annotated compounds were placed on the molecular network (Fig 6), putative structures were based on MS and MS/MS spectra and correspond to the first hit in the FragHub database.

**Table 1:** List of 38 m/z features detected and annotated (level 2) in *Rubus* leaves.

n°	RT (min)	m/z	Formula MW	fragments (m/z)	Annotation	SuperClass	Literature occurrence	Cluster
**1**	11.37	[M+H-2H_2_O]^+^ 469.3292	C_30_H_48_O_6_ 504.7	451, 433, 423, 405 (100), 213, 201, 187, 173, 159, 145, 133, 119	Hydroxytormentic acid or isomer derivatives	Triterpenoids	[25]	C1
**2**	10.43	[M+H-H_2_O]^+^ 455.3142	C_30_H_48_O_4_ 472.7	455 (100), 437, 409, 391, 203, 201, 187, 173, 159, 147, 133, 119	Pomolic acid or isomer	Triterpenoids	[25,40]	C1
**3**	10.82	[M+H-H_2_O]^+^ 455.3143	C_30_H_48_O_4_ 472.7	455, 437, 419, 409 (100), 391, 326, 213, 201, 187, 175, 145				
**4**	10.69	[M+H-H_2_O]^+^ 455.3142	C_30_H_48_O_4_ 472.7	455, 437, 409, 391 (100), 229, 215, 203, 187, 147, 133, 119				
**5**	12.07	[M-H_2_O+H]^+^ 471.3447	C_30_H_46_O_4_ 488.7	471, 453, 425, 407, 353, 219, 205, 201 (100), 187, 175, 147, 119	Tormentic acid or isomer	Triterpenoids	[25,40]	C1
**6**	12.16	[M-H_2_O+H]^+^ 471.3431	C_30_H_46_O_4_ 488.7	453, 425, 407, 205, 201 (100), 187, 177, 145, 119				
**7**	11.50	[M-H2O+H]^+^ 487.3387	C_30_H_48_O6 504.7	487, 469, 451, 441, 433, 423 (100), 405, 249, 191, 187, 159, 147	Hydroxytormentic acid or isomer	Triterpenoids	[25]	C1
**8**	11.90	[M-H_2_O+H]^+^ 487.3394	C_30_H_48_O6 504.7	487, 469, 451, 441 (100), 423, 405, 307, 249, 203, 187, 159				
**9**	12.45	[M-H_2_O+H]^+^ 487.3401	C_30_H_48_O6 504.7	487, 469, 451, 431, 423, 405 (100), 249, 245, 201, 187, 159, 147, 119				
**10**	11.31	[M-H]^–^ 501.1388	C_30_H_46_O_6_ 502.3	483 (100), 455, 441, 421	Ilexgenin A1 isomer	Triterpenoids	[25,41]	C1
	11.22	[M+H]^+^ 503.3338		503, 486, 485, 469, 455 (100), 421, 349, 249, 191, 187				
**11**	11.64	[M-H]^–^ 501.3212	C_30_H_46_O_6_ 502.3	501 (100), 485, 483, 453				
	11.60	[M+H]^+^ 503.3350		503 (100), 485, 467, 457, 421, 281, 251, 233, 215, 205, 187, 173				
**12**	12.12	[M-H]^–^ 501.3210	C_30_H_46_O_6_ 502.3	501 (100), 483, 465, 455 439, 421, 378, 247, 219				
	12.16	[M+H]^+^ 503.3353		503, 485, 457, 439 (100), 421, 415, 403, 304				
**13**	11.09	[M+NH_4_]^+^ 668.4356	C_36_H_58_O_10_ 650.8	490, 471, 453, 425, 407, 219, 215, 205 (100), 201, 187	Rosamultin or isomer	Triterpenoid glycosides	[41,42]	C1
**14**	10.74	[M+NH_4_]^+^ 682.4142	C_36_H_56_O_11_ 664.8	682, 676, 648, 503, 485, 467, 457, 449, 439 (100), 421, 403, 201, 187, 173, 159, 145	Ilexsaponin A1 or isomer	Triterpenoid glycosides	[43,44]	C1
**15**	10.44	[M+NH_4_]^+^ 684.4307	C_36_H_58_O_11_ 666.8	684, 631, 505, 487, 469 (100), 451, 423, 405, 249, 239, 213, 205, 201, 187, 173, 156	Dotorioside II or isomer	Triterpenoid glycosides	[25,27,42,45]	C1
**16**	10.62	[M+NH_4_]^+^ 684.4287	C_36_H_58_O_11_ 666.8	684, 505, 487, 469 (100), 451, 423, 405, 205, 201, 187				
**17**	10.81	[M+NH_4_]^+^ 684.4274	C_36_H_58_O_11_ 666.8	505, 487, 469, 451 (100), 441, 433, 423, 405, 229, 213, 201, 187, 173				
**18**	10.64	[M+H]^+^ 325.0921	C_15_H_16_O_8_ 324.28	163 (100), 145, 135, 117	Skimmin or isomer	Coumarin glycosides	[46]	C2a
**19**	0.87	[M+Na]^+^ 365.1021	C_12_H_22_O_11_ 342.30	365 (100), 244, 203, 185	Disaccharides	Saccharides	–	C2a
**20**	0.98	[M+Na]^+^ 365.1017	C_12_H_22_O_11_ 342.30	365 (100), 304, 203, 185, 104	Sucrose or isomer	Saccharides	–	C2a
**21**	10.91	[M]^+^ 463.1221	C_22_H_23_O_11_^+^ 462.4	463, 301 (100)	Peonidin 3-O-glucoside	Flavonoid glycosides	[47,48]	C2a
**22**	8.27	[M+H-H_2_O]^+^ 499.1224	C_25_H_24_O_12_ 516.04	499, 320, 163 (100)	Cinnamic acids and derivatives	Phenylpropanoids	[49]	C2a
**23**	9.47	[M+Na]^+^ 705.3793	C_36_H_58_O_12_ 682.8	705 (100), 543, 490	Oleane triterpenoids glycoside	Triterpenoid glycosides	–	C2a
**24**	7.86	[M+H]^+^ 463.0858	C_21_H_18_O_12_ 462.4	287 (100)	Kaempferol 3-O-glucuronide	Flavonoid glycosides	[50,51]	C2b
**25**	6.98	[M+H]^+^ 479.0804	C_21_H_18_O_13_ 478.4	303 (100)	Quercetin 3-O-glucuronide	Flavonoid glycosides	[52]	C2b
**26**	7.76	[M+H]^+^ 551.1015	C_24_H_22_O_15_ 550.4	303 (100)	Quercetin 3-o-beta-D-(6’‘-o-malonyl)-glucoside or isomer	Flavonoid glycosides	[53–55]	C2b
**27**	8.25	[M-H]^+^ 591.1337	C_27_H_28_O_15_ 592.5	591, 529, 489, 447, 285, 284 (100), 255, 277	Kaempferol 3-O-[6”-O-(3- hydroxy-3- methylglutaryl)]hexoside	Flavonoid glycosides	[56,57]	C2b
	8.28	[M+H]^+^ 593.149		287 (100)				
**28**	10.46	[M+H]^+^ 595.1437	C_30_H_26_O_13_ 594.5	287, 147 (100), 119	Tiliroside	Flavonoid glycosides	[58,59]	C2b
**29**	7.87	[M-H]^–^ 607.131	C_27_H_28_O_16_ 608.1	607, 545, 505, 463, 301, 300 (100)	Quercetin 3-O-[6”- O-(3-hydroxy-3- methylglutaryl)]hexoside	Flavonoid glycosides	[60]	C2b
	7.88	[M+H]^+^ 609.1440		303 (100)				
**30**	7.73	[M-H]^–^ 753.1867	C_33_H_38_O_20_ 754.6	753, 651, 609, 301, 300 (100)	Flavonol glycoside	Flavonoid glycosides	[61,62]	C2b
	7.75	[M+H]^+^ 755.2001		449, 345, 303 (100)				
**31**	10.75	[M-H]^–^ 285.0376	C_15_H_10_O_6_ 286.24	285 (100), 258, 239, 229, 211, 201, 187, 171, 159, 143	Kaempferol	Flavonoids	[60,63,64]	C6
	10.56	[M+H]^+^ 287.0537		287 (100), 258, 241, 213, 185, 157, 153, 121				
**32**	5.64	[M-H]^–^ 300.9966	C_14_H_6_O_8_ 302.197	301, 283, 257, 245, 229, 216, 201, 185, 173, 145, 129, 117	Ellagic acid	Flavonoids	[65–68]	C6
	5.88	[M+H]^+^ 303.0117		303, 275, 269, 247, 229, 201, 175, 163, 146, 93				
**33**	9.94	[M-H]^–^ 301.0325	C_15_H_10_O_7_ 302.23	301 (100), 273, 256, 213, 178, 151, 121, 107, 93	Quercetin	Flavonoids	[60,69–71]	C6
	10.00	[M+H]^+^ 303.0483		303 (100), 285, 257, 247, 229, 201, 183, 165, 153, 137				
**34**	10.46	[M+H]^+^ 643.2381	C_33_H_38_O_13_ 642.65	643, 625, 417, 386, 355, 241, 201, 181 (100), 167	Furfuranoid lignans	Lignans	–	C8
**35**	1.95	[M-H]^–^ 783.0655	C_34_H_24_O_22_ 784.54	783, 772, 635, 633, 615, 613, 597, 481, 463, 445, 377, 301, 275, 263, 248, 219	Gallotannins	Tannins	[72–75]	C13
	1.95	[M+NH_4_]^+^ 802.1078		786, 785, 465, 447, 405, 303, 277 (100), 259, 182				
**36**	7.83	[M+H-H_2_O]^+^ 331.1527	C_19_H_24_O_6_ 348.4	331 (100), 313, 287, 227, 189, 151, 1337, 119	Emophilane sesquiterpenoids	Sesquiterpenoids	[76,77]	C16
**37**	8.64	[M+Na]^+^ 547.2136	C_26_H_36_O_11_ 524.6	547 (100), 401, 350, 159	Neolignans glycoside	Lignans	[78,79]	C17
**38**	8.53	[M+Na]^+^ 547.2137	C_26_H_36_O_11_ 524.6	547 (100), 401, 350	Neolignans glycoside	Lignans		C17

Cluster C1 contained 76 ions detected between 9.47 and 12.69 min, all matching the triterpenoid superclass. Seventeen compounds with a C30 skeleton were annotated (Table 1), based on fragmentation patterns adapted from Gradillas et al. [25] (S2-S3 Figs.). Three main ursane-type classes were identified:

1. Tetrahydroxyurs-12-en-28-oic acid isomers (C_30_H_48_O_6_, mw: 504.32 Da) and derivatives: hydroxytormentic acid or isomers derivatives (**1**); hydroxytormentic acid or isomers (**7**, **8**, **9**); dotorioside II or isomers (**15**, **16**, **17**). Hexoside moieties produced a parent ion at *m/z* 684 [M + NH_4_]^+^ (**15**, **16**, **17**). Compound (**17**) showed additional MS/MS fragments compared to (**15**) and (**16**), suggesting a slightly different structure (S3 Fig.). Putative names were assigned based on reported occurrence in *Rubus* [25,27,42].
2. Trihydroxyurs-12-en-28-oic acid isomers (C_30_H_48_O_5_, mw: 488.35 Da): tormentic acid or isomers (**5**, **6**) [25,40]; rosamultin or isomers (**13**) [41,42].
3. Dihydroxyurs-12-en-28-oic acid isomers (C_30_H_48_O_4_, mw: 472.36 Da): pomolic acid or isomers (**2**, **3**, **4**) [25,40].

Additional triterpenoids were annotated outside these classes. Compounds (**10**, **11**, **12**) were annotated as ilexgenin A1 (C_30_H_46_O_6_, mw: 502.33 Da) based on database matching and shared *m/z* 485 and 467 fragments [25], confirmed by negative-mode MS/MS (Table 1). Two other ions with the same database match were not detected in negative mode and were considered 19α-hydroxyursolic acid-type aglycones [25,41]. Compound (**14**) matched ilexsaponin A1 (C_36_H_56_O_11_, mw: 664.82 Da [M + NH_4_]^+^), reported in *Ilex pubescens* roots [43,44]. The diversity of ursane-type triterpenoids and glycoside isomers detected here is consistent with previous reports in blackberry leaves [25].

The upper part of cluster C2 (C2a, Fig 6) contained glycosylated compounds from multiple superclasses —flavonoids, phenylpropanoids (including coumarins) and triterpenoids — linked by sugar moiety losses (Table 1). Among them, **18** (skimmin or isomer) and **22** (cinnamic acid derivative) are reported here for the first time in *Rubus* leaves.

The lower part of cluster C2 (C2b, Fig 6) contained flavonoid glycosides. Quercetin and kaempferol aglycones were identified via *m*/*z* 303.05 and 287.05 peaks, consistent with *Rubus* literature [60,69,81]. Seven compounds were annotated: quercetin 3-O-glucuronide (**25**, C_21_H_18_O_13_, *m/z* 478.0804 [M + H]^+^); quercetin 3-O-β-D-(6’‘-O-malonyl)-glucoside or isomer (**26**, C_24_H_22_O_15_, *m/z* 551.1015 [M + H]^+^); quercetin 3-O-[6“-O-(3-hydroxy-3-methylglutaryl)]hexoside (**29**, C_27_H_28_O_16_, *m/z* 609.1440 [M+H]^+^); kaempferol 3-O-glucuronide (**24**, C_21_H_18_O_12_, *m/z* 463.0858 [M+H]^+^); kaempferol 3-O-[6”-O-(3-hydroxy-3-methylglutaryl)]hexoside (**27**, C_27_H_28_O_15_, *m/z* 593.1490 [M + H]^+^), reported here for the first time in *Rubus*; tiliroside (**28**, C_30_H_26_O_13_, *m/z* 595.1437 [M + H]^+^). Remaining cluster C2 ions showed *m/z* 303.05 or 287.05 fragments and sugar losses of 146 and 162 Da, suggesting additional kaempferol or quercetin hexoside derivatives.

Cluster C6 contained kaempferol and quercetin aglycones arising from in-source fragmentation of C2b glycosides (S3 Fig.), as well as ellagic acid (**32**, *m/z* 303.0117 [M + H]^+^, C_14_H_6_O_8_) [65,68].

Lignans were annotated in clusters C8 and C17. Compound **34** (cluster C8) was annotated as a furfuranoid lignan (C_33_H_38_O_13_, *m/z* 643.2381 [M + H]^+^), matching HHDP-syringaresinol, first reported as a natural product in *Ficus hirta* [82], and not previously described in *Rubus* leaves. Remaining C8 nodes are derivatives or in-source fragments of **34**, showing characteristic neutral losses of pentose (132 Da), methyl (30 Da) and hydroxyl (34 Da) groups. Compounds **37** and **38** (cluster C17, *m/z* 547.2136 and 547.2137 [M + Na]^+^) were annotated as neolignan glycoside isomers (C_26_H_36_O_11_), reported here for the first time in *Rubus*. Ion at *m*/*z* 577.2229 from cluster C17 corresponded to the same structure with an additional methoxy group (C_27_H_38_O_12_). Related neolignan glycosides have been reported in *Epimedium sagittatum* and *Geum japonicum* [83,84].

Compound **35** (cluster C13, *m/z* 802.1078 [M + Na]^+^) was annotated as a gallotannin (C_34_H_24_O_22_), confirmed by [M − H]^−^ at *m/z* 783.0655. MS/MS fragments at *m/z* 303 and 277 (ESI+) and *m/z* 481, 301 and 275 (ESI−) are characteristic of this compound family [72–75]. A fragmentation pathway is proposed in supplementary materials (S3 Fig.). The seven other cluster C13 nodes were annotated as hydrolysable tannin derivatives: castalagin or vescalagin (C_41_H_26_O_26_) derivatives [74,75,85]; casuarictin (C_41_H_28_O_26_) isomers [73,86–89]; geraniin (C_41_H_28_O_27_) isomers [86,90]; sanguiin H1-10 derivatives or hippophaenin B (C_48_H_32_O_31_) isomer [91,92] (S3 Fig.). Such gallotannins have been reported in *Rubus* leaves, fruits and seeds [93–96].

Compound **36** (cluster C16, *m/z* 331.15) was annotated as an eremophilane sesquiterpenoid (C_19_H_24_O_6_, exact mass: 348.15 Da), detected as [M + H − H_2_O]^+^ due to in-source water loss [97]. Two isomeric compounds with identical MS/MS peaks (*m/z* 331, 313, 287, 255, 189, 151) have been reported in *Camellia japonica* and *Casearia arborea* [76,77], but to our knowledge not previously in *Rubus*.

Cluster C5 ions could not be properly annotated. They shared an MS/MS fragment at *m/z* 274 and displayed even measured masses, suggesting the presence of an even number of nitrogen atoms or NH_4_^+^ adducts.

### Biomarkers

Very Important for Projection (VIP) ions scores explaining the differences between groups have been extracted and listed for the first two components considering a significancy threshold of 1 according to the literature [98].

**Fig 7:**
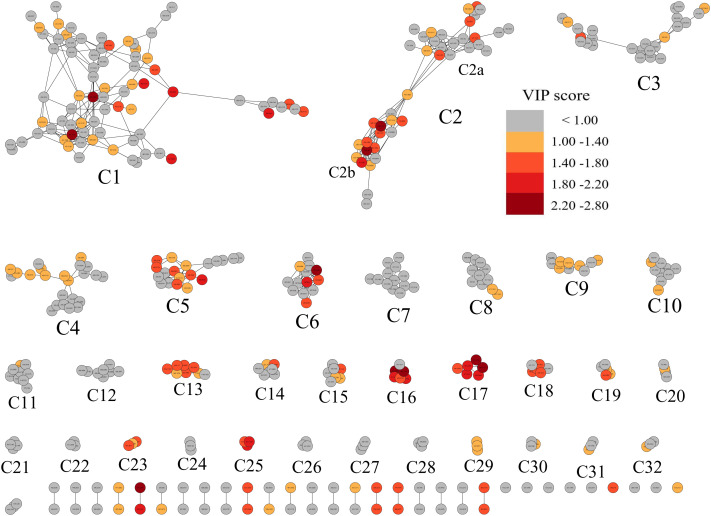
Molecular network based on MS/MS fragmentation by LC-MS ESI Q-TOF performed on *Rubus* leaf extracts. Color represents VIP’s scores extracted from first component of PLS-DA. Only the 9 first single nodes out of 279 were represented.

VIP scores and relative repartition of the three groups (*ID, C, RC*) were represented in the molecular network (Fig 7). Table 2 shows a summary of the main annotated compound’s VIP scores and statistics tests output.

**Table 2:** Spectral information and statistic outputs of VIP extraction completed by Anova/post-hoc tests of the of main cluster’s ion: Score VIP ranking out of the 668 matrice’s features (col 1); Tukey’s post hoc test output: different letters mean significative difference between groups with “a” being higher than “b” than “c” in terms of average ionic intensity (col 7,8,9); annotation (col 10); na means “not annotated”, ISF means “In Source Fragment”.

VIP Rank (/668)	VIP Score	Measured mass (*m/z*)	Row retention time (min)	Cluster	p-value Anova	Post-hoc test			Annotation
ID	C	RC
1	2,8553	547.2137	8.5314	C17	< 0.001	c	b	a	Compound **38**
2	2,7884	609.144	7.8772	C2b	< 0.001	a	b	b	Compound **29**
4	2,6993	547.2136	8.6448	C17	< 0.001	c	b	a	Compound **37**
5	2,6749	593.149	8.2789	C2b	< 0.001	a	b	b	Compound **27**
6	2,4838	469.3302	10.4329	C1	< 0.001	c	b	a	**15** ISF
10	2,3758	303.0487	7.8599	C6	< 0.001	a	b	b	**24** aglycone
11	2,3273	493.2208	10.6326	C16	< 0.001	c	b	a	**36** glc deriv. ISF
14	2,2395	451.3197	10.4336	C1	< 0.001	c	b	a	**15** ISF
15	2,2381	493.2199	9.9379	C16	< 0.001	c	b	a	na
18	2,1689	563.2082	8.973	C17	< 0.001	b	a	a	Neolignan glycoside
20	2,1129	475.2102	10.6282	C16	< 0.001	c	b	a	**36** glc deriv. ISF
21	2,0962	287.054	8.2951	C6	< 0.001	a	b	b	**27** aglycone
23	2,0156	611.248	9.6197	C2b	< 0.001	c	b	a	Flavonoid glycoside
25	1,9799	486.3048	8.4467	C5	< 0.001	a	b	b	na
27	1,9747	577.2229	9.5926	C17	< 0.001	c	a	b	Neolignan glycoside
28	1,9614	631.3829	10.4356	C1	< 0.001	c	b	a	**15** ISF
33	1,9359	511.2335	10.6309	C16	< 0.001	c	b	a	**36** glycoside derivative
34	1,9333	405.3135	10.4328	C1	< 0.001	c	b	a	**15** ISF
35	1,9087	700.4243	10.0927	C1	< 0.001	c	b	a	na
37	1,9003	684.4307	10.4366	C1	< 0.001	c	b	a	Compound **15**
38	1,8986	611.244	10.3753	C17	< 0.001	c	b	a	Neolignan glycoside
40	1,7998	953.1081	4.9904	C13	< 0.001	c	b	a	Gallotannins
42	1,7944	487.3407	10.4325	C1	< 0.001	c	b	a	**15** ISF
43	1,7923	500.2837	4.8256	C5	< 0.001	a	b	b	na
51	1,7427	486.3048	8.3545	C5	< 0.001	a	b	b	na
52	1,7349	533.2395	10.677	C2a	< 0.001	c	b	a	na
55	1,6964	482.2728	8.5481	C5	< 0.001	a	b	b	na
56	1,6905	641.2562	10.3643	C17	< 0.001	c	b	a	Neolignan glycoside
57	1,6857	953.6097	3.2156	C13	< 0.001	c	b	a	Gallotannins
63	1,6305	530.3301	10.3308	C5	< 0.001	a	b	b	na
65	1,6195	493.2193	10.1782	C16	< 0.001	c	b	a	na
68	1,5957	482.2724	8.4146	C5	< 0.001	a	b	b	na
70	1,5941	593.1484	8.6989	C2b	< 0.001	a	b	b	Flavonoid glycoside
71	1,5909	593.1485	8.0286	C2b	< 0.001	a	b	b	Flavonoid glycoside
73	1,5809	609.1433	8.4223	C2b	< 0.001	a	b	b	Flavonoid glycoside
80	1,5555	705.3793	9.4739	C2a	< 0.001	c	b	a	Compound **23**
81	1,5326	684.4287	10.6178	C1	< 0.001	c	b	a	Compound **16**
83	1,5254	325.0921	10.6477	C2a	< 0.001	b	a	a	Compound **18**
84	1,5131	303.0483	8.4147	C6	< 0.001	a	b	b	Quercetin aglycone
88	1,4763	682.4142	9.4736	C1	< 0.001	c	b	a	Compound **14**
89	1,4655	954.1169	7.4109	C13	< 0.001	b	a	a	Gallotannins
92	1,4483	641.3127	11.6801	C2a	< 0.001	a	b	c	na
93	1,4449	954.117	4.0014	C13	< 0.001	c	b	a	Gallotannins
99	1,4287	287.0536	9.0579	C6	< 0.001	a	c	b	Kaempferol aglycone
101	1,4268	467.3135	9.4709	C1	< 0.001	c	b	a	**14** ISF
102	1,4257	609.1431	7.7251	C2b	< 0.001	a	b	b	**30** ISF
106	1,4068	953.1074	0.7335	C13	< 0.001	b	a	a	Gallotannins
107	1,4047	755.2001	7.7458	C2b	< 0.001	a	b	b	Compound **30**
112	1,3846	465.1009	0.7254	C2b	< 0.001	a	b	c	Flavonoid glycoside
113	1,3835	482.2729	8.8847	C5	< 0.001	a	c	b	na
119	1,3501	451.3189	11.73	C1	< 0.001	a	b	b	na
120	1,3474	627.1539	0.7229	C2b	< 0.001	a	b	b	Flavonoid glycoside
123	1,3304	593.149	8.5508	C2b	< 0.001	a	b	b	Flavonoid glycoside
125	1,3223	463.0858	7.8618	C2b	< 0.001	b	b	a	Compound **24**
133	1,2988	469.3292	11.3674	C1	< 0.001	c	b	a	Compound **1**
138	1,2642	725.1882	8.0179	C2b	< 0.001	a	b	b	Flavonoid glycoside
143	1,2476	530.3299	10.4766	C5	< 0.001	a	b	b	na
148	1,2329	451.3182	11.3668	C1	< 0.001	b	b	a	**1** ISF
151	1,223	485.3232	11.603	C1	< 0.001	a	b	b	**11** ISF
162	1,1829	503.335	11.6016	C1	< 0.001	a	b	b	Compound **11**
168	1,1607	681.3824	8.4957	C2a	< 0.001	c	b	a	na
178	1,1312	469.3295	10.5976	C1	< 0.001	b	a	a	**16** ISF
180	1,1306	802.1078	1.9583	C13	< 0.001	b	a	a	Compound **35**
181	1,1306	451.3185	11.03	C1	< 0.001	c	b	a	na
182	1,1275	699.3541	12.3985	C2a	< 0.001	a	a	b	na
184	1,1206	512.3175	11.3045	C5	< 0.001	a	b	b	na
187	1,106	303.0486	7.2135	C6	< 0.001	a	b	b	Quercetin aglycone
199	1,0435	486.3049	8.5915	C5	< 0.001	a	b	b	na
201	1,039	1122.1217	5.1617	C13	< 0.001	b	a	a	Gallotannins
206	1,0296	451.3191	10.5973	C1	< 0.001	b	a	a	**16** ISF
210	1,0202	668.4356	11.0941	C1	< 0.001	b	a	a	Compound **13**
217	1,0024	677.3711	12.8885	C2a	< 0.001	a	a	b	na

Most of the highest-ranking VIPs were found in clusters C1, C2, C5, C6, C13, C16 and C17 (Table 2).

In cluster C1, 27 of 76 features were significant VIPs (Fig 7). Among triterpenoids, **15** (dotorioside II or isomer) was particularly discriminatory. This compound and related ursane-type triterpenoids have been reported in *Rubus* and other species for their bioactivities [99–101], and **15** appears to be a relevant biomarker of the *Corylifolii* and *Rubus* sections showing higher AIIs in the *RC* group (Table 2). Similarly, **14** (ilexsaponin A1 or isomers) and **1** (hydroxytormentic acid or isomers derivatives) were more represented in the *RC* group, whereas **11** (ilexgenin A1) was more characteristic of *R. idaeus* (Table 2). Compounds **13**, and **16**, and their in-source fragments also showed higher AIIs in the *RC* group (Table 2). The shared annotation of **15**, **16** and **17** likely reflects closely related structural isomers that could not be distinguished here. Still, AIIs’ trends of these three compounds revealed by tests showed divergences highlighting the importance of the numerous triterpenoids ursane type isomers. Overall, ursane-type triterpenoids appear as important contributors to *Rubus* taxa differentiation.

In cluster C16, all five significant VIPs (ranks 11, 15, 20, 33, 65), annotated as eremophilane-type sesquiterpenoids, followed a *RC* > *C* > *ID* AIIs’ gradient. These compounds are known for antibacterial and anti-inflammatory activities [102–104] and are, to our knowledge, reported here for the first time in *Rubus*.

In cluster C2, 20 of 40 features had significant VIP scores (Fig 7). Most flavonoid glycosides — including kaempferol 3-O-[6“-O-(3-hydroxy-3-methylglutaryl)]hexoside (**27**), quercetin 3-O-[6”-O-(3-hydroxy-3-methylglutaryl)]hexoside (**29**), compound **30** and their derivatives — were more abundant in the *ID* group, whereas kaempferol 3-O-glucuronide was more abundant in *RC* (Fig 7). Cluster C6 (quercetin and kaempferol aglycones, containing 5 significant ions) exhibited the same trend, as these ions largely arise from in-source fragmentation of C2 compounds. Compound **29** was especially associated with *R. idaeus*, as were **27**, **30**, and the kaempferol and quercetin moieties contained in C6 cluster. Flavonol glycosides acylated with 3-hydroxy-3-methylglutaric acid are known in *Rubus* for antioxidant, anti-inflammatory and antimicrobial activities [24,105,106] and have been described in *R. idaeus* and *R. fructicosus* cultivars [24,60,66]. Porter et al. [107] did not detect related compounds in *R. idaeus* or 12 other wild *Rubus* species, which may be explained by the promoting effect of alpine environments on many specialized metabolite production [108].

In cluster C13, 7 of 8 features had significant VIP scores (Fig 7). Compound **35** and most cluster ions showed lower AIIs in the *ID* group, with three ions (VIP ranks 40, 57 and 93) also differing between *RC* and *C*, suggesting that gallotannins are relevant markers for taxonomic classification (Table 2).

All six neolignan glycosides from cluster C17 were biomarkers of the *RC* group (Fig 7, Table 2). Compounds **37** and **38** had the highest VIP scores in the network (ranks 1 and 4) and followed a *RC* > *C* > *ID* AIIs’ gradient, as did most cluster ions, except ion at *m*/z 577.2229 (rank 27), which was higher in *C* than in *RC* (Table 2). Neolignan glycosides are known for protective roles against herbivores and microorganisms [109]. Xu et al. [110] identified five lignans (rubuslins) in *R. idaeus* leaves and rhizomes. Here, most annotated neolignan glycosides were more abundant in *Corylifolii*, *Rubus* and *Caesii* sections, and further work should confirm their value as section-level taxonomic markers.

In cluster C5, 10 of 19 features were significant VIPs, all more abundant in the *ID* group. These compounds could not be annotated and deserve further structural characterization.

Overall, results revealed a strong phytochemical structure across *Rubus* taxa. Three main chemical groups were identified: *R. idaeus*, *Rubus*/*Corylifolii*, and *Caesii*. *R. idaeus* was characterized by higher abundance of flavonol glycosides, while *Rubus*/*Corylifolii* were enriched in sesqui- and triterpenoids, neolignans and gallotannins. *Caesii* showed an intermediate profile, explaining the scarcity of clear biomarkers for this section. Further analyses are needed to identify markers at more specific taxonomic levels within these sections.

Using untargeted LC-QTOF/MS/MS metabolomics on *Rubus* leaf extracts from 9 alpine taxa, several polyphenols and terpenoids were putatively annotated as relevant taxonomic classifiers. This is one of the first attempts to validate *Rubus* taxonomy through phytochemical diversity. Triterpenoids, flavonoids, gallotannins and lignans have been previously reported in *Rubus* [24,25,60,66,93–96] but rarely compared across wild taxa for taxonomic purposes. Oszmiański et al. [66] analyzed fruit phenolics in 23 wild *Rubus* taxa and noted the lack of comparative chemical studies, detecting 34 phenolics without discussing their distribution. Our results support a more structured chemical differentiation. Eight compounds — coumarin glycoside (**18**), cinnamic acid derivatives (**22**), flavonoid glycosides (**27**, **30**), lignans (**34**, **37**, **38**) and sesquiterpenoid (**36**) — are reported here for the first time in *Rubus* leaves. This study provides a scientific basis for selecting *Rubus* taxa for targeted extraction of leaf compounds in a context of cosmetic or pharmaceutical valorization.

## Materials and methods

Part of the experiments, in particular plant sampling, were conducted in a natural zone located in Haute-Savoie (France). Field permit was delivered by Asters (Conservatoire Espaces Naturel Haute-Savoie) and an “Internationally recognized certificate of compliance constituted from information on the permit or its equivalent made available to the Access and Benefit-sharing Clearing-House” has been delivered by the United Nations Environment Program (ABSCH-IRCC-FR-259097–1).

### Sampling sites

The study area (around 4000 ha) is located within the limestone Tournette range (Massif des Bornes, Northern Alps, France) characterized by mostly calcareous soils and wet mountain climate with an average annual cumulated pluviometry of 1.7 m (Weather station Thônes, Les Besseaux (74), Alt. 630m, 1991–2020 data). After a comprehensive campaign led by the “Conservatoire d’Espaces Naturels de Haute-Savoie” (Asters) [111] ten stations (diameter 19 m) were sampled in 2023 based on their accessibility and the presence of *Rubus* L. specimens. In 2024, a new survey added 18 additional stations, to increase both the diversity of environmental conditions and the number of potential taxa. The 28 sites spread over an altitudinal gradient of 856 m to 1913 m represent several environmental habitats. Additionally, a station located on Clarins private production site named “Domain” (1153 m a.s.l.) was sampled because of its easier access to spontaneous *Rubus* L. specimens. Those specimens were used as references to monitor individual metabolome variation during the harvest season. Phytosociological and pedological descriptions of the stations are compiled in the supplementary (Table S2).

### Leaf sampling procedure

Two sampling campaigns were carried out in 2023 from the 5^th^ to 12^th^ September and in 2024 from the 20^th^ August to 17^th^ September. The taxa of interest were annotated as precisely as possible in the field using the criteria of different identification keys [5,12,112]. The term ‘morph’ used hereinafter refers to individuals with the same annotation result. Individuals of interest were counted and marked with a colored twine. To achieve robust sampling, 3–10 leaves depending on their size were collected from three to five individuals of the same morph at similar stages of development, i.e., after the growth peak period and before senescence. Before collecting the leaves, photographs of each individual were taken. *Rubus* have lignified stems adorned with prickles that can be divided into several ‘canes’ of different ages. The leaves were collected from the floricans (stems bearing inflorescences) and primocanes (leafy stems from the current year, also known as turions) and stored in chemically neutral paper bags. The samples were then dried in open air on racks for an average of ten days according to the protocol used at Clarins Laboratories. The 2023 sampling of 54 samples was carried out at 10 stations (10 stations x [3–8 replicates]). In 2024, the sampling was carried out at 28 stations, yielding to 192 samples (28 stations x [3–8 replicates]).

To provide a morphological reference and to confirm our identifications, one or more individual’s representative from the sample’s replicates intended for metabolomic analysis were collected to create an herbarium according to the protocol described in the literature [113]. These individuals were pressed and dried using newspaper and homemade press. The 2023 and 2024 herbarium contain respectively 21 and 53 specimens. Voucher specimens are available for consultation at the Grenoble Alpes University (Grenoble 38, France).

The presence of spontaneous *R. idaeus* at the ‘Domain’ site enabled weekly sampling in 2024 to monitor individual metabolome variation during the harvest season.

### Morphological annotations

Morphological annotations of the herbarium specimens were conducted using a botanical magnifying glass, following the criteria of various identification keys [4,5,12,112] and finally reviewed by David Mercier, Pascal Duboc and Jean-Marie Royer using both herbarium and field photographs. Some of the identifications must still be considered carefully due to the complexity of the genus and the poor documentation relative to *Rubus* of alpine mountains. Taxonomic ranks larger than species: subgenus, sections, subsection, series have then been used for some specimens. Morphological characters, including cane shape and pruinosity, armature type, leaf architecture, lower leaflet indumentum, presence or absence of stipitate glands, fruit colour and pruinosity were analyzed to annotate specimens. *Rubus idaeus* was readily identifiable by its pinnate leaves with a silvery-tomentose lower surface and its red fruits detaching freely from the receptacle. Distinction among the sections *Rubus*, *Hiemales*, *Corylifolii*, and *Caesii* was more challenging, because of the untypical aspects of alpine *Rubus* specimens collected for the present study. Section *Rubus* was identified according to the following main characters: digitately compound leaves, absence of stipitate glands, robust prickles, and black fruits remaining firmly attached to the receptacle. Presence of stipitate glands on stems and/or peduncles allowed to precise the identification to the *Hiemales* subsection. Section *Corylifolii* was identified according to the following main characters: often arching to sprawling habit, and ternate to digitately compound leaves. Specimens from *Caesii* section were identified according to the following main characters: stems and fruits bearing a conspicuous glaucous (pruinose) bloom; leaflets often ternate and lobed; fruits black and commonly pruinose. Detailed results of morphological identification are available in the supplementary data (Table S1). 2023 et 2024 samples were grouped in 5 and 9 taxonomic groups respectively (Table 3) based on the morphological analysis of each specimen and the taxonomic classification of *Rubus L.* to attribute homogenous taxonomic rank to the studied samples. Five groups based on morphological identification were considered in 2023: *ca* (*R. caesius* species); *Co* (*Coryfolii* section); *Co*_*Ru* (specimens that could not be distinguished from the *Rubus* and *Coryfolii* sections); *Hi* (*Hiemales* Subsection); *id* (*R. idaeus* species). The same five groups were found in 2024 sampling, along with four new groups: *Ca_Ru* (specimens that could not be distinguished from the *Caesi* and *Rubus* sections); *Co_Ru_ca* (specimens that could not be distinguished from the *Coryfolii* section, the *Pallidi* serie and the *caesius* species); *id Domain* (*R. idaeus* species sampled at the Domain station); *x* (specimens that couldn’t be identified) (Table 3, Fig 1). Fig 1B presents a simplified taxonomical *Rubus* scheme and the nine groups distribution.

**Table 3:** Sample groups based on morphological identifications of *Rubus* specimens collected in 2023 and 2024.

		2023		2024	
taxa	tax. Level	nb samples	nb station(s)	nb samples	nb station(s)
*ca*	specie	5	1	5	1
*Ca_Ru*	Section			3	1
*Co*	Section	5	1	4	1
*Co_Ru_ca*	Section_Serie_specie			4	1
*Co_Ru*	Section	5	1	3	1
*Hi*	Sub_Section	7*	2	61	16
*id*	specie	31	5	79	21
*id_D*	specie			30	1
*x*	NA			3	1

*Original number of samples was of 8 but one sample happened to be an outlier, and was therefore removed from the dataset explaining the total of 2023’s number samples of 53 than 54.

Details about morphological identifications are available in supplementary data and S1 Table.

### LC-MS/MS analysis

*Rubus* leaves were crushed to obtain pieces inferior to 0.5 mm. Extraction was carried out with a solvent (EtOH 80%) ratio to dry mass of 10 and an agitation-precession (Precellys ©) program with a rotation speed of 8000 rpm in 3 cycles of 15 seconds separated by 5-second breaks. The supernatant was filtered at 0.2 μm and then stored in vials in the dark at 8°C before being analyzed by UPLC-UV-ESI-QTOF. A biological pooled QC sample consisting of 10 μL of each sample extract was used to validate the repeatability of the metabolomics dataset and analyzed every ten runs during the whole sequence to constitute a technical replicate. Samples run order was randomly set using the function “RAND” from Microsoft Excel, except for the first four runs which were constituted by three blanks and then one QC to start the analysis. Liquid Chromatography was performed on Waters (Waters, Milford, Massachusetts, USA) Acquity I-Class UPLC with PDA coupled to a Waters Xevo G2-XS QTOF on a Thermoscientific C18 Hypersil Gold reverse phase column (2.1 x100 mm, particle size 1.9 µm). The chromatographic method used for the 2023 samples is as follows: Solvent A: H_2_O implemented with formic acid (0.1%) and ammonium formate (5 mM). Solvent B: methanol (100%). 0–0.5 min 5% B; 0.5–17 min 5- > 55% B; 17–20 min 55 - > 98% B; 20–28 min 98% B; 28–29 min 98%- > 5% B; 29–35 min 5% B. Flow rate: 0.4 μL/min. Mass spectrometer was set to perform Data Dependent Acquisition (DDA), with a detection window of 50–2000 Da, a MS detection threshold of 1000, and a scan time of 0.2 s. The MSMS parameters were programmed to fragment 3 ions, with a scan time of 0.2 s and a cumulative intensity threshold of 100,000. All spectra were acquired in continuum mode. A ramp was used to set the collision energy: 15–35 eV for molecules with a low molecular weight (50 Da) and 25–55 eV for molecules with a high molecular weight (1200 Da). All samples were analyzed in positive mode. Run time for each sample was 35 minutes. 2024 samples were analyzed with an optimized method based on the 2023 results. Time analysis was reduced to 22 minutes and a two steps gradient using acetonitrile instead of methanol appeared to be more suitable for the separation of compounds: 0–0.5 min 5% B; 0.5–1.5 min 5–10% B; 1.5–6 min - > 10% B; 6–7 min 10- > 20% B; 7–9 min 20% B; 9–13.5 min 20- > 98% B; 13.5–17.5 min 98% B; 17.5–20 min 98- > 5% B; 20–22 min 5% B. Flow rate: 0.4 μL/min. The DDA acquisition method was also optimized: detection window: 50–1200 Da, a MS detection threshold of 5 000, and a scan time of 0.2 s. The MSMS parameters were programmed to fragment 3 ions, with a scan time of 0.05 s and a cumulative intensity threshold of 10 000. All spectra were acquired in continuum mode. A ramp was used to set the collision energy: 5–40 eV for molecules with low molecular weight (50 Da) and 20–70 eV for molecules with high molecular weight (1200 Da). All samples were analyzed in positive mode. To verify the precision of our analysis during time, and the MS/MS detection, a quercetin standard provided by Extrasynthese (Genay, France) was analyzed every 10 runs. In addition, to assist with annotation, QC sample plus one sample of each taxonomic group were analyzed in negative mode using the same chromatographic and DDA parameters.

### Metabolomics data processing

Waters’ raw data were converted to mzML files using MSconvert (Version: 3.0.24124-ba8a4fd) and corrected by Precursor Corrector script (Elnur Garayev’s script, Windows_x64_v0.5) with the exact Lockspray’s Leucine *m*/*z* and the maximal error between precursors set by default. Data were processed on MZmine v.2.53 [114,115]. We used the MZmine 2 data-preprocessing workflow proposed by Olivon *et. al* [116] with a few adaptations to our data concerning the intensity threshold on the different steps. The 2023 et 2024 datasets were treated separately with their respective parameter adaptations. Ionic intensity (peak intensity) matrices of respectively 1322 and 1730 ions were generated for 2023 and 2024.

### Data analysis

MetaboAnalyst v.6.0 was used for statistical analysis preprocessing. Filters were used to remove features with low-quality, low repeatability based on QC samples (RSDs), and low variance based on interquantile range (IQR). Those filters were respectively set at thresholds of 80%, 55% and 40%. After this filtering step the 2023 and 2024 matrices contained respectively 566 and 668 features. Missing values have been replaced by 1/5 of the minimum positive value of the feature. The matrices were then centered and reduced using median normalization, cube root transformation and Pareto scaling algorithms to perform multivariate statistical analysis and analysis of variance (ANOVA). Filtered and normalized matrices were exported from MetaboAnalyst and imported on Rstudio (2025.9.1) running with R v.4.5.1. to conduct statistical analysis. The package Mixomics was used for multivariate analysis. First, Principal Component Analyses (PCA) was performed on both 2023 and 2024 matrices, using all biological samples, blanks, and Quality Controls to confirm the repeatability of the runs during the analysis (S4 Fig.). One sample from 2023 analysis belonging to the *Hi* group sampled at station 3 has been removed from the dataset because its PCA projection showed a trend that differed sharply from the rest of the group. Photographs of this sample were examined and compared with those of other replicas, and visual differences were noted in the appearance of the leaves (Fig. S5). The following multivariate analyses were performed on a dataset from which the QCs and blanks were removed. The most explanatory principal components were chosen to perform the PCA and Partial Least Squares Discriminant Analysis (PLS-DA). Dendrograms were calculated with the Dendextend package, using Ward’s clustering algorithms and Euclidean distance measures. VIP extraction has been realized on MetaboAnalyst v.06 by downloading the “Imp. Features” table.

### Molecular network and annotation

Molecular network representations were generated on MetGem V.1.5.2 from the MZmine ion intensity matrix containing only ions with associated MS2 fragmentation (106 samples x 668 ions). Despite duplicate and isotopes filtering during data processing, we observed the occurrence of duplicate ions and fragment nodes in the network, supposedly the result of in-source fragmentation. Choice has been made to keep those nodes in the network to conserve a maximum of spectral information to facilitate annotation. Still, potential in-source fragmentation phenomena have been carefully considered during nodes’ annotation process. Cosine score compilation parameters: *m*/*z* tolerance 0.02; minimum matched peaks: 2; Top K: 10; minimal cosine score value: 0.70; max. connected component size: 1000. An initial annotation attempt was made using spectral data libraries available on MetGem, an internal experimental library generated with standards, the Lotus library fragmented by FIORA, and the FragHub database downloaded in September 2025. Metgem’s libraries’ matches parameters were set as follow: *m*/*z* tolerance 0.02; Minimum matched peaks: 2; Minimum Cosine Score Values: 0.2. The matching parameters were voluntarily set loose to maximize the number of annotations. All outputs were then compared between the different databases and literature occurrences. Peak informations (molecular mass, MSMS fragmentation, retention time and UV absorbance) were also compared with literature. The molecular network displays have been produced on Cytoscape version 3.10.4 by loading the same.mgf file as the one used in Metgem.

## Supporting information

S1 Table Morphological identifications of *Rubus* specimens collected in 2023 and 2024. Content of columns from right to left: sampling station. Taxonomic identification and taxonomic rank; other possible identification; herbarium number.
Detailed morphological annotation below.
(DOCX)

S2 Table Site-specific and pedological characteristics of the stations. Erosion, soil moisture, soil compaction, and root abundance are discrete values represented on a five degrees scale: 0–5. Soil Texture: A/a: Clay, L/l: Loamy, S/s: sandy. PH was measured in water. Latitude and longitude are expressed in WGS84 (GPS).
(DOCX)

S1 Fig Complete molecular network (668 nodes) based on MS/MS fragmentation by LC-MS ESI Q-TOF performed on *Rubus* leaf extracts. Color tag is based on taxa groupment shown by multivariate analysis: purple = *ID* group; orange = *RC* group; red = *C* group, chart’s proportions were calculated by ionic intensity average of each feature in the three groups.
(DOCX)

S2 Fig Main triterpenoids complementary information. A: Triterpenoids focus on TIC chromatograms (RT: 9.20–12.65 min). Peaks annotated as triterpenoids are colored, and corresponding ions detected in MS1 are represented in the same color rectangle. Parent ions considered for annotation are bold later; in-source fragments are identified by *. Compounds number appear in parentheses. B: Annotated compounds’ putative structures. C: Fragmentation pathway proposed for each represented structure adapted from Gradillas *et al.,* 2019 [27]. Dashed arrows indicate that proposed molecular formula and adduct weren’t in the original publication.
(DOCX)

S3 Fig Complementary spectral information of annotated compounds and molecular network propagation. 3.1: Cluster C1. 3.2: Triterpenes from the tetrahydroxyurs-12-en-28-oic acid isomer class; compound 7, compound 8, compound 9, compound 15, compound 16, compound 17. 3.3: Triterpenes from the trihydroxyurs-12-en-28-oic acid class; compound 5, compound 13. 3.4: Triterpenes from the trihydroxyurs-12-ene-23(or24),28-dioic acid isomer class: compound 2, compound 3, compound 4. 3.5: Spectral data of triterpenes from ilexgenin A1 and ilexsaponin A1 isomers and derivatives; compound 11, compound 10, compound 12, compound 14. 3.6: Compound 18. S3.7: Clusters C6, C2b. S3.8: Compound 31, compound 32, compound 33. S3.9: Compound 24, compound 25, compound 26, compound 27. S3.10: Cluster C17. S3.11: Compound 38. S3.12: Cluster C13. S3.13: Compound 35. S3.14: Cluster C16. S3.15: Compound 36.
(DOCX)

S4 Fig Principal Component Analysis of the ionic intensity matrices figuring QC samples from the LC-MS ESI Q-TOF analysis of *Rubus* leaves: sampled in A: 2023 (53 samples; 21 QC x 566 features) and B: 2024 (192 samples; 26 QC x 668 features). (DOCX)

S5 Fig Supporting information concerning the removing of the outlier sample from 2023’s dataset. A: Principal Component Analysis of the ionic intensity matrices from the LC-MS ESI Q-TOF analysis of *Rubus* leaves sampled in 2023 (54 samples; 21 QC x 566 features), the red arrow identifies the outlier sample that has been removed. B: Photographs of the outlier sample. C: Photographs of a replica from the same taxonomic group sampled at the same station as the outlier sample. Comparison of photographs B and C reveals differences in the color of the upper and lower surfaces, the overall shape, and the dissection of the leaf blades.
(DOCX)

S6 Fig Graphical abstract. (JPG)
