# Supplementary material for: Untargeted metabolomics as a new perspective for assessing taxonomic boundaries in alpine Rubus genus
Source: PLoS One. 2026 Aug 5;21(8):e0354840. doi: 10.1371/journal.pone.0354840 (PMC13440846; doi:10.1371/journal.pone.0354840)
Supplement: Supplementary file 1 [file pone.0354840.s001.docx]

**S1 Table**: **Morphological identifications of *Rubus* specimens collected in 2023 and 2024**: content of columns from right to left: sampling station ; taxonomic identification and taxonomic rank; other possible identification; herbarium number. Detailed morphological annotation below.

| **Year** | **2023** | | | | **2024** | | | |
| --- | --- | --- | --- | --- | --- | --- | --- | --- |
| **Sampled station** | **Identification** | **Taxonomic rank** | **Alternative identification** | **herbarium n°** | **Identification** | **Taxonomic rank** | **Alternative identification** | **herbarium n°** |
| Station 1 |  |  |  |  | *R. vestitus* ;  Ser. *Vestiti* | Species | Ser. *Discolores* | *GR014021* |
| Station 3 | Ser. *Discolores* | Series | *R. mercieri* | *GR014001 ; GR014002 ; GR014003* | *R.* *vestitus* ;  Ser. *Vestiti* | Species |  | *GR014055* |
| station 5 |  |  |  |  | Sect. *Corylifolii* | Section | *R. Caesius* ; *R*. s*altuum* | *GR014047 ; GR014048* |
|  |  |  |  |  | *R. saltuum* ;  Ser. *Pallidi* | Species |  | *GR014035* |
| Station 13 | *R. idaeus* subsp. *Idaeus* | Subspecies |  | *GR014015 ; GR014017* | *R. idaeus* subsp. *Idaeus* | Subspecies |  | *GR014057 ; GR014025 ; GR014026 ;GR014027* |
|  |  |  |  |  | Ser. *Glandulosi* | Series |  | *GR014024* |
| Station 14 | Ser. *Glandulosi* | Series |  | *GR014020* | *R. saltuum* ;  Ser. *Pallidi* | Species |  | *GR014023* |
|  |  |  |  |  | *R. idaeus* subsp. *Idaeus* | Subspecies |  | *GR014058 ; GR014059* |
| Station 17 | Sect. *Corylifolii* | Section | Ser. *Subradula* ; Ser. *Hystricopses* | *GR014004* | Ser. *Micantes* | Series |  | *GR014042* |
| Station 18 | *R. idaeus* subsp. *Idaeus* | Subspecies |  | *GR014012 ; GR014013* | *R. idaeus* subsp. *Idaeus* | Subspecies |  | *GR014034* |
| Station 20 | Sect. *Corylifolii* | Section | Ser *Subradula* ; Sect. *Rubus* Ser. *Pallidi* | *GR014006 ; GR014007* | Sect. *Corylifolii* | Section |  | *GR014022* |
| Station 22 | *R. idaeus* subsp. *Idaeus* | Subspecies |  | *GR014008 ; GR014009* | *R. idaeus* subsp. *Idaeus* | Subspecies |  | *GR014032* |
| Station 25 | *R. idaeus* subsp. *Idaeus* | Subspecies |  | *GR014005* | *R. idaeus* subsp. *Idaeus* | Subspecies |  |  |
|  |  |  |  |  | *R. caesius* | Species | Ser. *Pallidi* | *GR014040* |
|  |  |  |  |  | Ser*. Pallidi* | Series | Sect*. Corylifolii* | *GR014041* |
| Station 27 | *R. caesius* | Species |  | *GR014018 ; GR014019* | *R. caesius* | Species |  | *GR014056* |
|  |  |  |  |  | *R. idaeus* subsp. *Idaeus* | Subspecies |  | *GR014045* |
| Station 33 | *R. idaeus* subsp. *Idaeus* | Subspecies |  | *GR014010 ; GR014011* | *R. idaeus* subsp. *Idaeus* | Subspecies |  | *GR014039* |
|  |  |  |  |  | Ser. *Glandulosi* | Series |  | *GR014049* |

**S1 Table**: ***suite***

| Station 130 |  |  |  |  | Ser*. Pallidi* | Series |  | *GR014031* |
| --- | --- | --- | --- | --- | --- | --- | --- | --- |
|  |  |  |  |  | *R. idaeus* subsp. *Idaeus* | Subspecies |  |  |
| Station 131 |  |  |  |  | Ser*. Pallidi* | Series |  | *GR014030* |
|  |  |  |  |  | *R. idaeus* subsp. *Idaeus* | Subspecies |  |  |
| Station 132 |  |  |  |  | *R. sp* | *Na* |  | *x* |
|  |  |  |  |  | *R. idaeus* subsp. *Idaeus* | Subspecies |  |  |
| Station 133 |  |  |  |  | Ser*. Pallidi* | Series | Ser. *Glandulosi* | *GR014029* |
|  |  |  |  |  | *R. idaeus* subsp. *Idaeus* | Subspecies |  |  |
| Station 134 |  |  |  |  | Ser*. Pallidi* | Series | Ser. *Glandulosi* | *GR014046* |
|  |  |  |  |  | *R. idaeus* subsp. *Idaeus* | Subspecies |  |  |
|  |  |  |  |  | Ser. *Glandulosi* | Series |  | *GR014052* |
| Station 135 |  |  |  |  | Ser*. Pallidi* | Series | Ser. *Glandulosi* | *GR01453 ; GR014054* |
|  |  |  |  |  | *R. idaeus* subsp. *Idaeus* | Subspecies |  |  |
| Station 136 |  |  |  |  | *R. idaeus* subsp. *Idaeus* | Subspecies |  | *GR014028* |
| Station 220 |  |  |  |  | *R. idaeus* subsp. *Idaeus* | Subspecies |  | *GR014033* |
| Station 300 |  |  |  |  | Ser*. Pallidi* | Series |  | *GR014038* |
|  |  |  |  |  | *R. idaeus* subsp. *Idaeus* | Subspecies |  |  |
| Station 301 |  |  |  |  | Ser. *Glandulosi* | Series |  | *GR014037* |
|  |  |  |  |  | *R. idaeus* subsp. *Idaeus* | Subspecies |  |  |
| Station 302 |  |  |  |  | *R. idaeus* subsp. *Idaeus* | Subspecies |  | *GR014036* |
| Station 420 |  |  |  |  | *R. idaeus* subsp. *Idaeus* | Subspecies |  | *GR014044* |
| Station 422 |  |  |  |  | *R. idaeus* subsp. *Idaeus* | Subspecies |  | *GR014043* |
| Station 430 |  |  |  |  | *R. idaeus* subsp. *Idaeus* | Subspecies |  | *GR014050* |
| Station 431 |  |  |  |  | Ser*. Pallidi* | Series | Ser. *Sprengeliani* ; Ser. *Micantes* | *GR014051* |

2023:

The 2023 Herbarium includes 10 specimens sampled on 10 stations.

-*id* group

5 specimens (from stations number 13, 18, 22, 25, 33 have been identified as *R. idaeus* subsp. *Idaeus* (*GR014015, GR014017, GR014012, GR014013, GR014008, GR014009, GR014005, GR014010, GR014011*) and attributed to the taxonomic group *id* (Table 1, Figure 1).

-*Co* group

One specimen from station 17 (*GR014004*) was described as belonging to the section *Corylifolii* subg. *Glaucobatus* with doubts remaining between the series *Hystricopses* and *Subradula* and was attributed to the taxonomic group *Co* (Table 1, Figure 1).

-*Co_Ru* group

One specimen from station 20 (*GR014006, GR014007*) was also described as belonging to section *Corylifolii* but an alternative belonging to series *Pallidi* (subg. *Rubus* sect. *Rubus* subsect. *Hiemales*) cannot be disregarded; it was consequently attributed to *Co_Ru* taxonomic group (Table 1, Figure 1).

-*Hi* group

Specimen from stations 14 (*GR014020*) was identified as belonging to the serie *Glandulosi* (subg. *Rubus* sect. *Rubus* subsect. *Hiemales*) and attributed to the *Hi* group.

One specimen from station 3, was characterized by a very large number of *Rubus* shrubs. It has been considered safer to sample 3 specimens for the same replicate series (*GR014001,* *GR014002,* *GR014003*). All of them were identified as belonging to the *Discolores* (*Piletocaulon*) *series* (subg. *Rubus* sect. *Rubus* subsect. *Hiemales*) but seems to be different species. *GR014001 was* identified as *R. mercieri* whereas samples number *GR014002* and *GR014003* were described as 2 distinct unidentified species belonging to *the Discolores* (*Piletaucolon*) series and consequently joined the *Hi* group (Table 1, Figure 1).

-*ca* group

 Specimen station 27 (*GR014018*, *GR014019*) were identified as belonging to the species *R. caesius* (Subg. *Glaucobatus*, Sect. *Caesi*) and attributed to the *ca* groups (Table 1, Figure 1).

2024:

The 2024 Herbarium includes 43 specimens. 10 of the 43 were collected from the same stations as in 2023 and the 33 others were collected from 18 new stations.

**Results relevant with 2023’s identification**

*-id* group

The presence of *R. idaeus* Subsp. *Idaeus* at stations 13 ; 18 ; 22 ; 25 ; 33 was consistent with 2023 results.

-*Co* group

The specimen *GR014022* sampled at station 20 was attributed to the section *Corylifolii* according to the 2023 results and have consequently been classed in the *Co* group.

*Hi* group

The specimen *GR014021* sampled at station 3 were assigned to *Hi* group, with doubts remaining with the series *Discolores* (*Piletocaulon*) (subg. *Rubus* sect. *Rubus* subsect. *Hiemales*) as proposed for the 2023 herbarium.

-*ca* group

Sample from station 27 (*GR014056*) was identified as *R. caesius* at in accordance with the result obtained for 2023 herbarium and was also attributed to *ca* group (Table 1, Figure 1).

**Additional results**

*-id* group

In addition, *R. idaeus* was also found on 16 supplementary stations (14 ; 27 ; 130 ; 131 ; 132 ; 133 ; 134 ; 135 ; 136 ; 220 ; 300 ; 301 ; 302 ; 420 ; 422 ; 430). Among them 2 were already studied in 2023 (14 ; 27) for other *Rubus* taxa and at the time the presence of *R. idaeus* had not been noticed. Those were attributed to *id* group.

-*Hi* group

Specimen station 1 (*GR014021*) has been identified as *R. vestitus* (subg. *Rubus* sect. *Rubus* subsect. *Hiemales* ser. *Vestiti*) and assigned to *Hi* group.

The presence of a different *Rubus* taxa at station 13 had been noticed and sampled (*GR014024*). The morphological identification indicates the presence of the series *Glandulosi* (subg. *Rubus* sect. *Rubus* subsect. *Hiemales*). Same conclusions have been made regarding the specimens from stations 33 (*GR014049*) ; 134 (*GR014052*) and 301 (*GR014037*). Those samples were attributed to the group *Hi* (Table 1, Figure 1).

Serie *Pallidi* has been assigned to samples from the stations 5 (precisely the species *R. saltuum*, *GR014035*), 14 (precisely the species *R. saltuum*, *GR014023*), 130 (*GR014031*), 131 (*GR014030*), 133 (*GR014029*), 134 (*GR014046*), 135 (*GR014053* and *GR014054*), 431 (*GR014051*). Those were all attributed to *Hi* group (Table 1, Figure 1) even if among those results some doubts remain for the specimens *GR014029*, *GR014046,* *GR01453*, *GR014054* with series *Glandulosi* (subg. *Rubus* sect. *Rubus* subsect. *Hiemales*). The specimen *GR014051* also raised doubts with the series *Sprengeliani* and *Micantes* (both belonging to subg. *Rubus* sect. *Rubus* subsect. *Hiemales*).

It can also be noticed that the *Rubus* taxa from station 14 was identified as *Glandulosi* series in 2023 (*GR014025*).

Differences of identification results between 2023 and 2024 can be observed at station 17. In 2023 the collected specimen has been identified as belonging to the section *Corylifolii* (*GR014004*) whereas in 2024 the identification led to assigning the sample to the series *Micantes* (subg. *Rubus* sect. *Rubus* subsect. *Hiemales*) (*GR014042*) and therefore assigned to *Hi* group.

-*Co_Ru* group

Taxa *GR014041* from station 25 was identified as belonging to the series *Pallidi* (subg. *Rubus* sect. *Rubus* subsect. *Hiemales*), also with doubts concerning the *Corylifolii* section (Subg. *Glaucobatus*) and was attributed to the *Co_Ru* group (Table 1, Figure 1).

-*Ca_Ru* group

Taxa *GR014040* from station 25 was identified as *R. caesius* (Subg. *Glaucobatus*, Sect. *Caesi*) with some doubts remaining with *Pallidi* series (subg. *Rubus* sect. *Rubus* subsect. *Hiemales*) and has been consequently attributed to the *Ca_Ru* group (Table 1, Figure 1).

-*Co_Ru_ca* group

At station 5, the specimen *GR014047* and *GR014048* were identified as belonging to section *Corylifolii* (Subg. *Glaucobatus*) with doubts remaining concerning the species *R. caesius* (Subg. *Glaucobatus*, Sect. *Caesi*) and *R. saltuum* (subg. *Rubus* sect. *Rubus* subsect. *Hiemales* ser. *Pallidi*) and was consequently attributed to *Co_Ru_ca* group (Table 1, Figure 1).

*-x* group

Finally, the specimen sampled station 132 (*x*) couldn’t be identified and was consequently attributed to *x* group.
