# Supplementary material for: Untargeted metabolomics as a new perspective for assessing taxonomic boundaries in alpine Rubus genus
Source: PLoS One. 2026 Aug 5;21(8):e0354840. doi: 10.1371/journal.pone.0354840 (PMC13440846; doi:10.1371/journal.pone.0354840)
Supplement: Supplementary file 2 [file pone.0354840.s002.docx]

**S2 Table:** **Site-specific and pedological characteristics of the stations.** Erosion, soil moisture, soil compaction, and root abundance are discrete values represented on a five degrees scale: 0 - 5. Soil Texture: A/a: Clay, L/l: Loamy, S/s: sandy.  PH was measured in water. Latitude and longitude are expressed in WGS84 (GPS).

| **Station** | **Elevation (m)** | **Slope (°)** | **Exposure** | **Surface stone cover (%)** | **Erosion** | **Soil depth (cm)** | **Soil moisture** | **Texture class** | **Coarse fragments (%)** | **Soil compaction** | **Root abundance** | **soil pH** | **Latitude (°)** | **Longitude (°)** |
| --- | --- | --- | --- | --- | --- | --- | --- | --- | --- | --- | --- | --- | --- | --- |
| **1** | 1141 | 23 | SE | 0 | 0 | >40 | 3 | La | 0 | 3 | 3 | 7,5 | 45,79974 | 6,32284 |
| **3** | 856 | 24 | E | 0 | 0 | >40 | 2 | L | 50 | 2 | 1 | 7 | 45,81931 | 6,33175 |
| **5** | 1212 | 15 | SE | 10 | 0 | >40 | 2 | La | 0 | 3 | 1 | 7 | 45,82314 | 6,26237 |
| **13** | 1524 | 19 | SE | 0 | 0 | >40 | 2 | La | 20 | 2 | 3 | 4,5 | 45,77651 | 6,28538 |
| **14** | 1562 | 39,6 | SE | 4 | 4 | 20 | 2 | La | 0 | 3 | 2 | 6 | 45,77957 | 6,28582 |
| **17** | 1208 | 36 | S | 0 | 4 | >40 | 4 | L | 20 | 1 | 2 | 6 | 45,81260 | 6,31459 |
| **18** | 1423 | 41 | SO | 40 | 5 | >40 | 2 | L | 0 | 3 | 2 | 4 | 45,82651 | 6,27030 |
| **20** | 1260 | 33 | SO | 0 | 5 | >40 | 1 | L | 0 | 2 | 2 | 7 | 45,81448 | 6,31213 |
| **22** | 1788 | 43 | SO | 80 | 5 | 10 | 1 | L | 70 | 4 | 3 | 7 | 45,83270 | 6,27454 |
| **25** | 1410 | 35 | E | 40 | 4 | 20 | 4 | L | 5 | 2 | 2 | 7 | 45,82003 | 6,30730 |
| **27** | 1166 | 52 | O | 60 | 5 | 7 | 2 | L | 70 | 4 | 1 | 7 | 45,81995 | 6,30988 |
| **33** | 1327 | 15 | NE | 10 | 4 | >40 | 2 | L | 35 | 3 | 2 | 6,5 | 45,84275 | 6,26998 |
| **130** | 1241 | 40 | NO | 5 | 2 | >40 | 3 | Als | 5 | 2 | 1 | 5 | 45,77726 | 6,27915 |
| **131** | 1322 | 41 | NO | 15 | 4 | >40 | 3 | Sl | 5 | 2,5 | 1 | 5,5 | 45,77741 | 6,28070 |
| **132** | 1317 | 28,6 | O | 40 | 4 | >40 | 2 | Sl | 15 | 2 | 2,5 | 5 | 45,77656 | 6,28116 |
| **133** | 1410 | 43,3 | S | 60 | 4 | >40 | Na | La | 40 | 2,5 | 2 | 6,5 | 45,77383 | 6,28198 |
| **134** | 1461 | 48 | S | 5 | 2 4 | >40 | 2 | La | 10 | 2 | 2 | 7 | 45,77339 | 6,28400 |
| **135** | 1535 | 32 | NO | 10 | 0 | >40 | 2 | Als | 15 | 2 | 1 | 4 | 45,77531 | 6,28409 |
| **136** | 1540 | 2 | SE | 0 | 1 | >40 | 2,5 | A | 20 | 1 | 3 | 4 | 45,77532 | 6,28455 |
| **220** | 1530 | 15,6 | NO | 5 | 1 | >40 | 2 | L | 5 | 2 | 3 | 4 | 45,83163 | 6,26782 |
| **300** | 1489 | 12,6 | SE | 5 | 1 | 35 | 2 | La | 25 | 2//3 | 2,5 | 6 | 45,81922 | 6,30500 |
| **301** | 1535 | 33,3 | E | 10 | 1 | >40 | 2,5 | La | 20 | 1 | 1 | 4,5 | 45,81849 | 6,30291 |
| **302** | 1611 | 33,3 | E | 10 | 1 | >40 | 2,5 | Sl | 10 | 2 | 2 | 5 | 45,81853 | 6,30096 |
| **420** | 1645 | 18,6 | S | 70 | 1 | 15 | 3 | Sl | 25 | 4 | 2,5 | 7 | 45,81782 | 6,28061 |
| **422** | 1763 | 5,6 | SO | 33 | 1 | 10 | 3 | Sl | 50 | 4 | 1 | 7 | 45,81465 | 6,28590 |
| **430** | 1907 | 23 | NO | 20 | 1 | >40 | 2,5 | Als | 20 | 2,5 | 2 | 4 | 45,81183 | 6,28969 |
| **431** | 1913 | 24,6 | O | 5 | 1 | >40 | 2 | Als | 5 | 1 | 2,5 | 4 | 45,81269 | 6,28986 |
| **Domaine** | 1153 | 28,3 | SO | 5 | 1 | >40 | 2 | La | 15 | 2 | 2 | 6 | 45,81250 | 6,31122 |
