# Supplementary material for: Untargeted metabolomics as a new perspective for assessing taxonomic boundaries in alpine Rubus genus
Source: PLoS One. 2026 Aug 5;21(8):e0354840. doi: 10.1371/journal.pone.0354840 (PMC13440846; doi:10.1371/journal.pone.0354840)
Supplement: Supplementary file 3 [file pone.0354840.s003.docx]

C32
**n=3**

C20
**n=4**

C10
**n=10**

C3
**n=25**

C33

C32

C31

C30

C29

C27

C28

C26

C25

C24

C23

C22

C20

C10

C5

C3

C21

C19

C18

C17

C16

C15

C14

C13

C12

C11

C9

C8

C7

C6

C4

C2

C2a

C2b

C1

**Figure S1:** Complete molecular network (668 nodes) based on MS/MS fragmentation by LC-MS ESI Q-TOF performed on *Rubus* leaf extracts. Color tag is based on taxa groupment shown by multivariate analysis : purple = *ID* group ; orange = *RC* group ; red = *C* group, chart’s proportions were calculated by ionic intensity average of each feature in the three groups.
