# Supplementary material for: Untargeted metabolomics as a new perspective for assessing taxonomic boundaries in alpine Rubus genus
Source: PLoS One. 2026 Aug 5;21(8):e0354840. doi: 10.1371/journal.pone.0354840 (PMC13440846; doi:10.1371/journal.pone.0354840)
Supplement: Supplementary file 4 [file pone.0354840.s004.docx]

**Figure S2: Main triterpenoids complementary information**. **A:** Triterpenoids focus on TIC (Total Ion Chromatograms) (RT: 9.20 – 12.65min). Peaks annotated as triterpenoids are colored, and corresponding ions detected in MS1 are represented in the same color rectangle. Parent ions considered for annotation are bold later; in-source fragments are identified by *. Compounds number appear in parentheses. **B:** Annotated compounds’ putative structures. **C:** Fragmentation pathway proposed for each represented structure adapted from Gradillas *et al.,* 2019*.* Dashed arrows indicate that proposed molecular formula and adduct weren’t in the original publication.
