# Supplementary material for: Untargeted metabolomics as a new perspective for assessing taxonomic boundaries in alpine Rubus genus
Source: PLoS One. 2026 Aug 5;21(8):e0354840. doi: 10.1371/journal.pone.0354840 (PMC13440846; doi:10.1371/journal.pone.0354840)
Supplement: Supplementary file 5 [file pone.0354840.s005.docx]

**S3.1: Focus on nodes from C1 cluster and their affiliation to triterpenes classes.**

**S3.2: MS2 spectra (ESI+) of triterpenes from the tetrahydroxyurs-12-en-28-oic acid isomer class: compound 7 (B), compound 8 (C), compound 9 (D), compound 15 (E), compound 16 (F), compound 17 (G).**

Compounds from the tetrahydroxyurs-12-en-28-oic acid isomer class: **1**, **7**, **8**, **9**, **15**, **16** and **17** shared MS/MS and MS in source fragmentation corresponding to a C_30_H_48_O_6_ (504.34 Da) ursane-type skeleton. Acid and consecutive water losses produced the main observed fragmentation peaks. Compound C_30_H_46_O_6_ after a water loss [M-H_2_O+H]^+^ produced a measured mass at *m*/*z* 487 (**7**, **8**, **9**). Consecutive losses of water produced ions *m*/*z* 469 [M-2H_2_O+H]^+^ (**1,7**), *m*/*z* 451 [M-3H_2_O+H]^+^ (**1**, **8**, **9**, **15**, **16**, **17**) and *m*/*z* 433 [M-4H_2_O+H]^+^ (**1**, **17**). Additional losses of acid function produced the fragments *m*/*z* 441 [M-COOH-H_2_O+H]^+^ (**7**, **17**), *m*/*z* 423 [M-HCOOH-2H_2_O+H]^+^ (**1**, **7**, **8**, **9**, **15**, **16**, **17**), and *m*/*z* 405 [M-HCOOH-3H_2_O+H]^+^ (**1**, **7**, **8**, **9**, **15**, **16**, **17**). Hexoside moieties produced a parent ion at *m*/*z* 684 corresponding to a [M+NH_4_]^+^ (**15**, **16**, **17**). MS/MS peaks measured in the spectra of the previously cited compounds sometimes presented 2 Da gaps suggesting structural variation such as double bonds consistent with the wide numbers of structural isomers in the triterpenoid family. This led to the annotation of the following compounds: hydroxytormentic acid or isomers derivatives (**1**) ; hydroxytormentic acid or isomers (**7**, **8**, **9**) ; dotorioside II or isomers (**15**, **16**, **17**). By examining the fragments generated for the compounds **15**, **16** and **17**, we could observe more fragments in the MS/MS spectra of compound **17**, suggesting a slightly different structure than those of compounds **15** and **16**.

**S3.3:** **MS2 spectra (ESI+) of triterpenes from the trihydroxyurs-12-en-28-oic acid class:** compound **5 (A),** compound **13 (B).**

Compounds from the trihydroxyurs-12-en-28-oic acid isomer class (**5, 6, 13**) shared MS/MS fragments and MS in source fragmentation corresponding to a C_30_H_46_O_5_ (488.3502 Da) ursanes-type skeleton. Water losses produced the main observed fragmentation peaks and suggested annotation of the following compounds: tormentic acid or isomers (**5**, **6**) ; rosamultin or isomers (**13**). Additionally, three ions from the cluster with characteristic MS/MS fragments (*m*/*z* 407, 201) have been annotated as derivatives from the previous structures: *m/z* 453.3325 (RT: 11.30 min), *m*/*z* 453.3332 (RT: 12.43 min) and *m*/*z* 453.3343 (RT: 12.61 min) (S4.1).

 **S3.4 : MS2 spectra (ESI+) of triterpenes from the trihydroxyurs-12-ene-23(or24),28-dioic acid isomer class:** compound **2 (A),** compound **3 (B),** compound **4 (C).**

Compounds from the trihydroxyurs-12-ene-23(or24),28-dioc acid isomer class (**2, 3, 4**) shared MS/MS fragments and MS in source fragmentation corresponding to a C_30_H_48_O_4_ (472.3553 Da) ursane-type skeleton. Water losses produced the main observed fragmentation peaks. The following annotation of: pomolic acid or isomers (**2**, **3**, **4**) was proposed.

 **S3.5 : Spectral data of triterpenes from ilexgenin A1 and ilexsaponin A1 isomers and derivatives:** compound **11 (A)** top MS2 ESI+, bottom MS2 ESI−; compound **10** MS2 ESI+ **(B)**; in-source fragmentation (MS1) ESI+ of compound **12 (C)**; compound **12** MS2 ESI− **(D)** ; compound **14** MS2 ESI+ **(E).**

Three ions with close measured masses *m*/*z* 503.3338 (RT: 12.45 min), *m*/*z* 503.3350 (RT:11.60 min) and *m*/*z* 503.3353 (RT: 12.16 min) were detected. They all matched in the spectral databases with ilexgenin A_1_ (C_30_H_46_O_6_, exact mass: 502.3294) and shared *m*/*z* 485 and 467 fragments. Compounds **10**, **11**, **12** were therefore annotated as ilexgenin A_1_ after MS/MS in negative mode confirmation. Two other ions from the cluster have the same database match as **10**, **11** and **12**, but weren’t detected in negative mode. They have been considered as triterpenoids belonging to the same series: 19α-hydroxyursolic acid-type aglycone. Compound 1**4** (main fragment *m*/*z* 457.3289) matched in databases with a glycoside ursane triterpenoid (C_36_H_56_O_11_), named ilexsaponin A1. We found an ion with the measured mass *m*/*z* 467.3236 with close retention time as compound **14**, suggesting in-source fragmentation. Three ions with close measured masses: *m*/*z* 682.4141 (RT: 10.74 min), *m*/*z* 682.4132 (RT: 12.09 min) et *m*/*z* 682.4129 (RT: 10.99 min) sharing fragmentation peaks with compound **14** and matching with ilexsaponin A1 in spectral databases were therefore annotated as isomers.

**S3.6: Putative spectral mass fragmentation (MSMS) of compound 18** m/z 325.0921 [M+H]^+^ ; retention time 10.64 min after alignment and the chemical putative structure obtained with top matching score of FragHub database.

The upper part of the C2 cluster matched with compounds belonging to different superclasses, such as coumarins, flavonoids, phenylpropanoids and triterpenoids linked in the cluster by the losses of sugar moieties, suggesting that they are all glycosylated compounds. For example, **21** annotated as peonidin 3-O-glucoside (C_22_H_23_O_11_^+^ ; *m/z* 463.1221 [M]^+^), and **23** annotated as an oleane triterpenoid glycoside (C_36_H_58_O_12_ ; *m/z* 705.3793 [M+Na]^+^), showed MSMS peaks at 301 and 543 respectively, both corresponding to a hexose loss. The other nodes of the cluster (including compounds **18**, **19**, **20**, **22**) also presented hexose losses in their MS/MS spectra (-162 Da) explaining that they have been clustered together.

 **S3.7: Parent ions and in-source fragments of glycosylated flavonoid derivatives (cluster C2b) and kaempferol, quercetin and ellagic acid aglycones (cluster C6).** The color code corresponds to the different aglycones. The measured mass in *m/z* and the retention time in minutes (absolute) are indicated within the nodes. The numbers of annotated compounds are shown in parentheses, parent ions are in bold and fragment ions in regular characters.

 **S3.8: MS2 spectra in positive and negative mode of kaempferol, ellagic acid and quercetin detected in cluster 6. A:** compound **31** (kaempferol), ESI+ (top), ESI− (bottom) ; **B**: compound **32** (ellagic acid), ESI+ (top), ESI− (bottom) ; **C**: compound **33** (quercetin), ESI+ (top), ESI− (bottom). The proposed fragmentation pathways are adapted from literature (Table 1).

 **S3.9: Spectral data of glycosylated flavonoids from cluster C2b:** compound **24** MS2 ESI+ (A); compound **25** MS2 ESI+ (B); compound **26** MS2 ESI+ (C); compound **27** (D) top MS2 ESI+, bottom MS2 ESI−.

The cluster C6 contained several other nodes that matched kaempferol and quercetin in the spectral databases with *m/z* 287.05 or 303.04 and similar MS/MS fragmentation but with different retention times. Those nodes correspond to aglycons of kaempferol and quercetin glycoside derivatives found in cluster C2 due to in-source fragmentation. For example, the node from cluster C6 at *m/z* 303.0496 has a retention time of 7.01 min, close to quercetin 3-O-glucuronide (**25**) retention time (6.98 min). In addition to these compounds ellagic acid (measured mass of *m/z* 303.0117 [M+H]^+^, molecular formula C_14_H_6_O_8_) was found in the cluster (**32**). Fragments at *m/z* 257 [M-H-CO_2_]^-^ and 229 [M-H-CO_2_-CO]^-^ (negative mode) corresponded to the fragmentation pathway of ellagic acid.

**S3.10**: **Focus on nodes from C17 cluster.** The measured masses in *m/z* are indicated within the nodes.

**S3.11: MS2 spectrum in positive mode of compound 38.**

Compounds **37** (*m/z* 547.2136 [M+Na]^+^), **38** (*m/z* 547.2137 [M+Na]^+^) were annotated in cluster C17 as isomers of neolignan glycosides (C_26_H_36_O_11_) differing in their deoxyhexose moiety. Ion with the measured mass at *m/z* 577.2229 corresponded to neolignan glycosides **37** and **38** with an additional methoxy group, leading to the molecular formula C_27_H_38_O_12_. Other ions from the cluster detected at *m*/*z* 643.2389 (RT: 10.72 min) and *m*/*z* 643.24 (RT: 10.99 min) showed the same fragments: 417, 385, 181 and 167 matched with the same compound from databases, suggesting the presence of isomers from this compound.

**S3.12:** **Focus on the 8 ions of cluster C13 and the putative annotations performed by propagation.** The measured mass in *m/z* and the retention time in minutes (absolute) are indicated within the nodes.

**S3.13: Spectral data and fragmentation pathway of compound 35. A:** MS2 spectrum in ESI+ (top) and ESI− (bottom) mode. **B:** Fragmentation pathway adapted from literature (Table 1). Fragments found in the MS2+ and MS2− spectra were annotated in italics and in parentheses and reported on the spectra.

**Compound 35** (*m/z* 802.1078, [M+Na]^+^; C_34_H_24_O_22_) was annotated as a gallotannin, supported by the [M−H]^−^ peak at *m/z* 783.0655 in negative mode. MS2 fragments at *m/z* 481, 301 and 275 (negative mode) are characteristic of ellagitannins, specifically pedunculagin (C_34_H_24_O_22_; exact mass: 784.54 Da). The *m/z* 481 fragment corresponds to an HHDP-glucose moiety (fragment *g2*; Fig. S4.13) after HHDP loss [M−302], *m/z* 301 to an HHDP residue (fragment *g3*) after HHDP-hexose loss [M−482], and *m/z* 275 to a C_13_H_7_O_7_^−^ fragment (fragment *g4*) after HHDP decarboxylation.

The remaining seven nodes of cluster C13 (Fig. S4.12) shared the *m/z* 303.01 fragment (ellagic acid, positive-mode MS2) and *m/z* 453.01 (glucosyl residue loss, characteristic of gallotannins). The *m/z* 615 fragment observed for the heaviest ion (*m/z* 1150.0118) is consistent with castalagin or vescalagin (C_41_H_26_O_26_) fragmentation after HHDP and water losses, though insufficient data prevented definitive assignment.

Two ions at *m/z* 954.1169 (RT = 7.41 min) and *m/z* 954.1170 (RT = 4.00 min) were annotated as casuarictin isomers (C_41_H_28_O_26_) based on negative-mode MS2 peaks at *m/z* 633, 300 and 275, consistent with reported fragmentation. The retention time difference (~3 min) suggests distinct structures.

Three ions at *m/z* 953.1074 (RT = 0.73 min), *m/z* 953.1081 (RT = 4.99 min) and *m/z* 953.6097 (RT = 3.22 min) showed positive-mode MS2 peaks at *m/z* 783, 303 and 277, consistent with geraniin (C_41_H_28_O_27_) fragmentation (reported at *m/z* 781, 301, 275 in negative mode). Mass differences (up to 0.5 Da) and retention time variability suggest these are distinct isomers or derivatives, possibly trisgalloyl HHDP-glucose or granatin B.

The *m/z* 1103 ion is tentatively assigned to hippophaenin B (C_48_H_32_O_31_) based on positive-mode MS2 peaks at *m/z* 935, 785 and 635, consistent with reported negative-mode fragments (*m/z* 1059, 935, 783, 633), confirming its belonging to the hydrolysable tannin family.

**S3.14 Figure: Focus on the 6 ions of cluster C16 and the putative annotations performed by propagation.** The measured mass in *m/z* and the retention time in minutes (absolute) are indicated within the nodes.

**S3.15 Figure:** **MS2 spectrum in positive mode of compound 36.**

Compound **36** from cluster C16, *m*/*z* 331.15, was annotated as an eremophilane sesquiterpenoids according to databases matches: C_19_H_24_O_6_ (exact mass: 348.15 Da) suggesting a precursor ion [M+H-H_2_O]^+^ due to in-source fragmentation leading to a water loss. Two isomeric compounds named 1,6-di-acetyl-furannoeremophilone and naphtho[2,3-b]furan-9(4H)-one,4,8- bis(acetyloxy) -4a,5,6,7,8,8a-hexahydro-3,4a,5-trimethyl-, (4S,4aR,5S,8S,8aS)- with the same MS/MS peaks: *m/z* 331, 313, 287, 255, 189 and 151 were found in literature.

The remaining ions from cluster C16 shared common MS/MS fragments such as *m/z* 351, 313, 287, 227, 151, and 137. Measured masses suggested that remaining ions from cluster C16 are derivatives from **36**: ion at *m/z* 511.2335 (RT: 10.63 min) (GD36) is more likely a glycoside derivative as indicated by the fragment at *m/z* 163. Ions at *m/z* 493.2208 and 475.2102 suggested two consecutive losses of water of the ion at *m/z* 511.2335 probably due to in-source fragmentation: all three having the same retention time (10.63 min).
