# Supplementary material for: Untargeted metabolomics as a new perspective for assessing taxonomic boundaries in alpine Rubus genus
Source: PLoS One. 2026 Aug 5;21(8):e0354840. doi: 10.1371/journal.pone.0354840 (PMC13440846; doi:10.1371/journal.pone.0354840)
Supplement: Supplementary file 6 [file pone.0354840.s006.docx]

**A.**

**B.**

**Figure S4:** Principal Component Analysis of the ionic intensity matrices figuring QC samples from the LC-MS ESI Q-TOF analysis of *Rubus* leaves sampled in **A**: 2023 (53 samples ; 21 QC x 566 features) and **B**: 2024 (192 samples ; 26 QC x 668 features).
