# Supplementary material for: Untargeted metabolomics as a new perspective for assessing taxonomic boundaries in alpine Rubus genus
Source: PLoS One. 2026 Aug 5;21(8):e0354840. doi: 10.1371/journal.pone.0354840 (PMC13440846; doi:10.1371/journal.pone.0354840)
Supplement: Supplementary file 7 [file pone.0354840.s007.docx]

**Figure S5:** Supporting information concerning the removing of the outlier sample from 2023’s dataset. **A**: Principal Component Analysis of the ionic intensity matrices from the LC-MS ESI Q-TOF analysis of *Rubus* leaves sampled in 2023 (54 samples ; 21 QC x 566 features), the red arrow identifies the outlier sample that has been removed. **B**: Photographs of the outlier sample. **C**: Photographs of a replica from the same taxonomic group sampled at the same station as the outlier sample. Comparison of photographs **B** and **C** reveals differences in the color of the upper and lower surfaces, the overall shape, and the dissection of the leaf blades.
