# Supplementary material for: Untargeted metabolomics as a new perspective for assessing taxonomic boundaries in alpine Rubus genus
Source: PLoS One. 2026 Aug 5;21(8):e0354840. doi: 10.1371/journal.pone.0354840 (PMC13440846; doi:10.1371/journal.pone.0354840)
Supplement: Supplementary file 8 [file pone.0354840.s010.docx]

Response to Reviewers

Article: **Untargeted metabolomics as a new perspective for assessing taxonomic boundaries in alpine *Rubus* genus.**

Corresponding author: **E. Serviole**

**Corrections apply to the manuscript are visible in the text as they are highlighted in yellow. When a whole part of the manuscript had to be modified, only the title of the concern part is highlighted in yellow to conserve readability.**

We detailed the modifications made on the submission files according to comments from the journal and comments from “reviewer #1” and “reviewer #2” respectively.

**Journal recommendations**:

- First, we wanted to clarify role of the different structures involved in the realization of this article. This work is part of a phD CIFRE contract (*Conventions Industrielles de Formation par la Recherche*) supervised by the collaboration of the Savoie Mont-Blanc University (public actor) and the Laboratoires Clarins (private actor). This collaboration is supervised and financed by the state organization ANRT (*Association Nationale de la Recherche et de la Technologie*).
- We confirm in **our Funding Statement** we’ll be update mentioning that : “**all the funding or sources of support (whether external or internal to your organization) received during this study, were provided by the Research and Technology National Association (ANRT) under the CIFRE project n°2022/1240. There was no additional external funding received for this study**.”
  This amendment will be included in the cover letter.
- “Fundings” part has been removed from the manuscript.
- Some authors are indeed **employed by** **Laboratoires Clarins SAS**. An amended Funding Statement declaring this commercial affiliation, we’ll be provided as well as a statement regarding the Role of Funders in your study. **The funding organization did play a role in the study design**, data collection and analysis, decision to publish, or preparation of the manuscript **and did not only provide financial support** in the form of authors' salaries and/or research materials. Roles of the authors were specifically and accurately indicated in our study.
- Considering your advice regarding the impact of data sharing plant on acceptance delay, it was decided to store the data in the following repository: [**https://github.com/e1000ser/Metabolomics-analysis-of-wild-Rubus-leaves**](https://github.com/e1000ser/Metabolomics-analysis-of-wild-Rubus-leaves) , hosted on the **GitHub platform**. Data availability statement of the submission form will be updated accordingly.
- **Antoine Henriot** participated in the work related to this article and should therefore appear as an author. However, the structure he’s affiliated to: ASTERS (*Conservatoire d'Espaces Naturels de Haute-Savoie*) which is the Haute-Savoie Nature Conservancy, do not appear in the affiliation options. After asking the PLOS customer care service, we were told to type it into the box manually without selecting an option from the dropdown.
- Figures 1, 4, 5 and 7 have been regenerated **as .TIFF files with better resolution**. Figures 5 and 7 show molecular networks and readers can therefore want to zoom in, so .svg files have also been proposed for those two figures.
- The last table of the manuscript was renamed “**Table 3**” instead of “Table 1”.
- **Full ethics statement** has been included in the ‘Methods’ section of the manuscript file **and** has been completed with the reference of the “**Internationally recognized certificate of compliance constituted from information on the permit or its equivalent made available to the Access and Benefit-sharing Clearing-House**” delivered by the “United Nations Environment Programme”.

**First, we would like to warmly thank the two reviewers for their comments, which greatly helped us improve this manuscript, particularly with regard to its length and structure.**

**Reviewer #1 recommendations**:

- Introduction has been considered too long, so the **number of words has been reduced from 874 to 587 words**. Because most of the sentences were restructured, we couldn’t annotate all the modifications made on the introduction and proposed it as a new text. However, **we kept the original’s organization as follows**, reducing each part:

- Global presentation of the genus (- 4 words)

- Taxonomic complexity resulting from the apomictical characteristics of the genus. (-67 words)

- Presentation of the actual state of *Rubus* taxonomy highlighting the divergences and instability of certain taxa. (-122 v2 words)

- A brief focus on the phytochemical compounds known in the genus as well as their bioactive propriety and utilization. (-39 words)

- Summary of publications using untargeted metabolomics as a tool to assess taxonomic problematics. (-55 words)

- **Morphologic characters used to identify the collected *Rubus*** specimens have been added **in the Materials and Method in the “Morphological annotation”** part. Because the morphological identification of *Rubus* taxa depends on a lot of morphological criteria (sometimes identification presents more than 50 morphological verifications for a single taxa) we proposed to **present only the main characters** which were different between the nine taxa identified. The whole morphological analysis can be provided if needed.
- **Precise location** of sampling sites has been added to **S2 table**.
- The **chapter on results** was found to be too long and have been therefore reduced, mostly by : 1- **Reducing the description of multivariate analysis’** figures and focusing on the shared tendances of PCA, PLS-DA, and HCA for both years. 2- **Reducing the description of metabolites fragmentation** and related spectral information. Those data were synthetized in the **supplementary figure S4**.
- **Ionization mode**: ESI has been precised at the beginning of the “Results” section as in the “LC-MS/MS analysis” section of the Materials and method.
- **Keys-words** have been added right after the abstract as recommended.
- Number of **citations has been reduced** as recommended **from 164 to 116**, mostly by reducing the citations in the annotation table. choosing the articles according to the following hierarchical points of pertinence: 1-articles that specifically describe the observed fragments 2- articles mentioning the compound in *Rubus* genus 3- most recent publication.

**Reviewer #2 recommendations**:

- **Righteousness of molecular formula**, and proposed structures have been verified. Some mistakes were found in S2 Figure (new numeration: see below) and have been corrected.
- Results and discussion have been regrouped in a single part **“Results and discussion”** to reduce the length of the manuscript and to clarify the message, as recommended.

Overall, new manuscript text is **5632 words long compared to 8464 words** in the first version.

**Additional modifications:**

- **Numeration of supplementary figures** has been reordered according to their apparition in text.

S1 Fig. became S4 Fig.

S2 Fig. became S1 Fig.

S3 Fig. became S2 Fig.

S4 Fig. became S3 Fig.

S5 Fig. stayed the same.

- **Number of samples collected in 2023** wrote in the “leaf sampling procedure section” was false and has been **corrected from 48 to 54**. Because one sample considered as an **outlier** was removed from the dataset the total of **samples from 2023 collected is 53**, a **note under Table 3** was added to explain the missing value.
- Mistake was found in the **fragmentation pathway of gallotannin** compound in the supplementary **figure S3 (new numeration)** and was corrected.
- A mistake has been found in Table 1 concerning **compound 14 retention time and MSMS fragments did not correspond to the spectrum**. Same mistake was found for **compound 33**. Both mistakes have been corrected.
- **“A” and “B”** letters were missing on **Figs. 2 and 3** and were therefore replaced by **“Left” and “Right”** in figures captions.
