# Supplementary material for: Untargeted metabolomics as a new perspective for assessing taxonomic boundaries in alpine Rubus genus
Source: PLoS One. 2026 Aug 5;21(8):e0354840. doi: 10.1371/journal.pone.0354840 (PMC13440846; doi:10.1371/journal.pone.0354840)
Supplement: Peer review history

# Peer review: Decision Letter 0

**DOI:** 10.1371/journal.pone.0354840.r001

**Authors:** Chun-Hua Wang

2 Jun 2026

PONE-D-26-18871

Untargeted metabolomics as a new perspective for assessing taxonomic boundaries in alpine Rubus genus.

PLOS One

Dear Dr. SERVIOLE,

Thank you for submitting your manuscript to PLOS ONE. After careful consideration, we feel that it has merit but does not fully meet PLOS ONE’s publication criteria as it currently stands. Therefore, we invite you to submit a revised version of the manuscript that addresses the points raised during the review process.

Please submit your revised manuscript by Jul 17 2026 11:59PM. If you will need more time than this to complete your revisions, please reply to this message or contact the journal office at plosone@plos.org. When you're ready to submit your revision, log on to https://www.editorialmanager.com/pone/ and select the 'Submissions Needing Revision' folder to locate your manuscript file.

Please include the following items when submitting your revised manuscript:

- A letter that responds to each point raised by the academic editor and reviewer(s). You should upload this letter as a separate file labeled 'Response to Reviewers'.
- A marked-up copy of your manuscript that highlights changes made to the original version. You should upload this as a separate file labeled 'Revised Manuscript with Track Changes'.
- An unmarked version of your revised paper without tracked changes. You should upload this as a separate file labeled 'Manuscript'.

If you would like to make changes to your financial disclosure, please include your updated statement in your cover letter. Guidelines for resubmitting your figure files are available below the reviewer comments at the end of this letter.

If applicable, we recommend that you deposit your laboratory protocols in protocols.io to enhance the reproducibility of your results. Protocols.io assigns your protocol its own identifier (DOI) so that it can be cited independently in the future. For instructions see: https://journals.plos.org/plosone/s/submission-guidelines#loc-laboratory-protocols. Additionally, PLOS ONE offers an option for publishing peer-reviewed Lab Protocol articles, which describe protocols hosted on protocols.io. Read more information on sharing protocols at https://plos.org/protocols?utm_medium=editorial-email&utm_source=authorletters&utm_campaign=protocols.

As the corresponding author, your ORCID iD is verified in the submission system and will appear in the published article. PLOS supports the use of ORCID, and we encourage all coauthors to register for an ORCID iD and use it as well. Please encourage your coauthors to verify their ORCID iD within the submission system before final acceptance, as unverified ORCID iDs will not appear in the published article. *Only*  the individual author can complete the verification step; PLOS staff *cannot*  verify ORCID iDs on behalf of authors.

We look forward to receiving your revised manuscript.

Kind regards,

Chun-Hua Wang

Academic Editor

PLOS One

Journal Requirements:

When submitting your revision, we need you to address these additional requirements.

1. Please ensure that your manuscript meets PLOS ONE's style requirements, including those for file naming. The PLOS ONE style templates can be found at https://journals.plos.org/plosone/s/file?id=wjVg/PLOSOne_formatting_sample_main_body.pdf and https://journals.plos.org/plosone/s/file?id=ba62/PLOSOne_formatting_sample_title_authors_affiliations.pdf

2. Thank you for stating in your Funding Statement:

[This work was supported by the research and Technology National Association (ANRT) under the CIFRE project n°2022/1240.].

Please provide an amended statement that declares *all* the funding or sources of support (whether external or internal to your organization) received during this study, as detailed online in our guide for authors at http://journals.plos.org/plosone/s/submit-now.  Please also include the statement “There was no additional external funding received for this study.” in your updated Funding Statement.

Please include your amended Funding Statement within your cover letter. We will change the online submission form on your behalf.

3. Please remove any funding-related text from the manuscript. Please note that funding information should not appear in any section or other areas of your manuscript. We will publish the funding statement that is present in the Financial Disclosure section of the online submission form.

4. Thank you for stating the following in the Competing Interests section:

[The authors have declared that no competing interests exist.].

We note that one or more of the authors are employed by a commercial company: Laboratoires Clarins SAS.

1. Please provide an amended Funding Statement declaring this commercial affiliation, as well as a statement regarding the Role of Funders in your study. If the funding organization did not play a role in the study design, data collection and analysis, decision to publish, or preparation of the manuscript and only provided financial support in the form of authors' salaries and/or research materials, please review your statements relating to the author contributions, and ensure you have specifically and accurately indicated the role(s) that these authors had in your study. You can update author roles in the Author Contributions section of the online submission form.

Please also include the following statement within your amended Funding Statement.

“The funder provided support in the form of salaries for authors [insert relevant initials], but did not have any additional role in the study design, data collection and analysis, decision to publish, or preparation of the manuscript. The specific roles of these authors are articulated in the ‘author contributions’ section.”

If your commercial affiliation did play a role in your study, please state and explain this role within your updated Funding Statement.

2. Please also provide an updated Competing Interests Statement declaring this commercial affiliation along with any other relevant declarations relating to employment, consultancy, patents, products in development, or marketed products, etc.

Within your Competing Interests Statement, please confirm that this commercial affiliation does not alter your adherence to all PLOS ONE policies on sharing data and materials by including the following statement: "This does not alter our adherence to PLOS ONE policies on sharing data and materials.” (as detailed online in our guide for authors http://journals.plos.org/plosone/s/competing-interests) . If this adherence statement is not accurate and there are restrictions on sharing of data and/or materials, please state these. Please note that we cannot proceed with consideration of your article until this information has been declared.

Please include both an updated Funding Statement and Competing Interests Statement in your cover letter. We will change the online submission form on your behalf.

5. When completing the data availability statement of the submission form, we note that you have provided the dataset source that is used for your study. However, the DOI that you provided shows “DOI not found”, which suggests that the DOI may not have been activated yet, and will be possibly available on acceptance. We strongly recommend all authors decide on a data sharing plan before acceptance, as the process can be lengthy and hold up publication timelines. Please note that, though access restrictions are acceptable now, your entire data will need to be made freely accessible if your manuscript is accepted for publication. This policy applies to all data except where public deposition would breach compliance with the protocol approved by your research ethics board. If you are unable to adhere to our open data policy, please kindly revise your statement to explain your reasoning and we will seek the editor's input on an exemption. Please be assured that, once you have provided your new statement, the assessment of your exemption will not hold up the peer review process.

6. Please amend the manuscript submission data (via Edit Submission) to include author Antoine Henriot.

7. Please include your full ethics statement in the ‘Methods’ section of your manuscript file. In your statement, please include the full name of the IRB or ethics committee who approved or waived your study, as well as whether or not you obtained informed written or verbal consent. If consent was waived for your study, please include this information in your statement as well.

8. Please upload a new copy of Figures 1, 4, 5, and 7 as the details are not clear. Please follow the link for more information:  https://journals.plos.org/plosone/s/figures

9. Please note that there are currently two tables labeled as "Table 1" on pages 10-13 and 32. Please renumber your tables and update the corresponding citations in the text to ensure they follow a sequential order.

If the reviewer comments include a recommendation to cite specific previously published works, please review and evaluate these publications to determine whether they are relevant and should be cited. There is no requirement to cite these works unless the editor has indicated otherwise.

Please review your reference list to ensure that it is complete and correct. If you have cited papers that have been retracted, please include the rationale for doing so in the manuscript text, or remove these references and replace them with relevant current references. Any changes to the reference list should be mentioned in the rebuttal letter that accompanies your revised manuscript. If you need to cite a retracted article, indicate the article’s retracted status in the References list and also include a citation and full reference for the retraction notice.

[Note: HTML markup is below. Please do not edit.]

Reviewers' comments:

Reviewer's Responses to Questions

**Comments to the Author**

1. Is the manuscript technically sound, and do the data support the conclusions?

The manuscript must describe a technically sound piece of scientific research with data that supports the conclusions. Experiments must have been conducted rigorously, with appropriate controls, replication, and sample sizes. The conclusions must be drawn appropriately based on the data presented.

Reviewer #1: Yes

Reviewer #2: Yes

**********

2. Has the statistical analysis been performed appropriately and rigorously?

Reviewer #1: Yes

Reviewer #2: Yes

**********

3. Have the authors made all data underlying the findings in their manuscript fully available?

The PLOS Data policy requires authors to make all data underlying the findings described in their manuscript fully available without restriction, with rare exception (please refer to the Data Availability Statement in the manuscript PDF file). The data should be provided as part of the manuscript or its supporting information, or deposited to a public repository. For example, in addition to summary statistics, the data points behind means, medians and variance measures should be available. If there are restrictions on publicly sharing data—e.g. participant privacy or use of data from a third party—those must be specified.

Reviewer #1: Yes

Reviewer #2: Yes

**********

4. Is the manuscript presented in an intelligible fashion and written in standard English?

PLOS ONE does not copyedit accepted manuscripts, so the language in submitted articles must be clear, correct, and unambiguous. Any typographical or grammatical errors should be corrected at revision, so please note any specific errors here.

Reviewer #1: Yes

Reviewer #2: Yes

**********

5. Review Comments to the Author

Please use the space provided to explain your answers to the questions above. You may also include additional comments for the author, including concerns about dual publication, research ethics, or publication ethics. (Please upload your review as an attachment if it exceeds 20,000 characters)

Reviewer #1: Dear Editor in Chief

PLOSE One

The manuscript entitled Untargeted Metabolomics

as a new perspective for assessing

taxonomic boundaries in alpine Rubus

Genus has been reviewed. The subject

is interesting and useful for readerships.

Some points need to be considered for

further clarification:

1- Introduction section is too long.

It should be summarized.

2- what is the scientific method

for identifying all taxa?

3- what are exact geographical statues

of collected samples?

4-Results section is too long.

It should be summarized and keep

key information.

5- What are two factors; CID and ESI modes?

6- Please add keywords at the end of abstract

section.

7- 164 references: should be considered

important and key references.

Accordingly, manuscript needs revision.

Sincerely Yours

Reviewer #2: The manuscript presents a very well-designed and executed study, certainly of great relevance in terms of the solid science of chemical ecology.

However, it contains some incomplete and unformatted references, chemical substances with typographical errors, or molecular formulas without subscripts.

As is common in this format, with the results separated from the discussion, the text becomes confusing. Please carefully review this separation to make it more appropriate. There are several loose and meaningless sentences that should be better connected to the text. Another text structure is suggested to better group the results and their discussion.

**********

6. PLOS authors have the option to publish the peer review history of their article (what does this mean?). If published, this will include your full peer review and any attached files.

If you choose “no”, your identity will remain anonymous but your review may still be made public.

**Do you want your identity to be public for this peer review?**  For information about this choice, including consent withdrawal, please see our Privacy Policy.

Reviewer #1: No

Reviewer #2: No

**********

[NOTE: If reviewer comments were submitted as an attachment file, they will be attached to this email and accessible via the submission site. Please log into your account, locate the manuscript record, and check for the action link "View Attachments". If this link does not appear, there are no attachment files.]

To ensure your figures meet our technical requirements, please review our figure guidelines: https://journals.plos.org/plosone/s/figures

You may also use PLOS’s free figure tool, NAAS, to help you prepare publication quality figures: https://journals.plos.org/plosone/s/figures#loc-tools-for-figure-preparation.

NAAS will assess whether your figures meet our technical requirements by comparing each figure against our figure specifications.

# Author comment: Author response to Decision Letter 1

**DOI:** 10.1371/journal.pone.0354840.r002

9 Jul 2026

Dear Editor, dear reviewvers,

We would like to warmly thank you for your comments, which greatly helped us improve this manuscript, particularly with regard to its length and structure.

All the suggested modifications have been described in the "response to the reviewers". Here's the main points :

Reviewer #1 recommendations:

• Introduction has been considered too long, so the number of words has been reduced from 874 to 587 words. Because most of the sentences were restructured, we couldn’t annotate all the modifications made on the introduction and proposed it as a new text. However, we kept the original’s organization as follows, reducing each part:

- Global presentation of the genus (- 4 words)

- Taxonomic complexity resulting from the apomictical characteristics of the genus. (-67 words)

- Presentation of the actual state of Rubus taxonomy highlighting the divergences and instability of certain taxa. (-122 v2 words)

- A brief focus on the phytochemical compounds known in the genus as well as their bioactive propriety and utilization. (-39 words)

- Summary of publications using untargeted metabolomics as a tool to assess taxonomic problematics. (-55 words)

• Morphologic characters used to identify the collected Rubus specimens have been added in the Materials and Method in the “Morphological annotation” part. Because the morphological identification of Rubus taxa depends on a lot of morphological criteria (sometimes identification presents more than 50 morphological verifications for a single taxa) we proposed to present only the main characters which were different between the nine taxa identified. The whole morphological analysis can be provided if needed.

• Precise location of sampling sites has been added to S2 table.

• The chapter on results was found to be too long and have been therefore reduced, mostly by : 1- Reducing the description of multivariate analysis’ figures and focusing on the shared tendances of PCA, PLS-DA, and HCA for both years. 2- Reducing the description of metabolites fragmentation and related spectral information. Those data were synthetized in the supplementary figure S4.

• Ionization mode: ESI has been precised at the beginning of the “Results” section as in the “LC-MS/MS analysis” section of the Materials and method.

• Keys-words have been added right after the abstract as recommended.

• Number of citations has been reduced as recommended from 164 to 116, mostly by reducing the citations in the annotation table. choosing the articles according to the following hierarchical points of pertinence: 1-articles that specifically describe the observed fragments 2- articles mentioning the compound in Rubus genus 3- most recent publication.

Reviewer #2 recommendations:

• Righteousness of molecular formula, and proposed structures have been verified. Some mistakes were found in S2 Figure (new numeration: see below) and have been corrected.

• Results and discussion have been regrouped in a single part “Results and discussion” to reduce the length of the manuscript and to clarify the message, as recommended.

Overall, new manuscript text is 5632 words long compared to 8464 words in the first version.

We hope you found this new version an improvement, and that we took care of the corrections you suggested as you intended.

We remain at your disposal, if any information or modification are needed.

Kind regards,

E. Serviole

Attachment Submitted filename: Response to Reviewers_ES.docx

# Editor report: Decision Letter 1

**DOI:** 10.1371/journal.pone.0354840.r003

**Authors:** Chun-Hua Wang

13 Jul 2026

Untargeted metabolomics as a new perspective for assessing taxonomic boundaries in alpine Rubus genus.

PONE-D-26-18871R1

Dear Dr. SERVIOLE,

We’re pleased to inform you that your manuscript has been judged scientifically suitable for publication and will be formally accepted for publication once it meets all outstanding technical requirements.

Within one week, you’ll receive an e-mail detailing the required amendments. When these have been addressed, you’ll receive a formal acceptance letter and your manuscript will be scheduled for publication.

An invoice will be generated when your article is formally accepted. Please note, if your institution has a publishing partnership with PLOS and your article meets the relevant criteria, all or part of your publication costs will be covered. Please make sure your user information is up-to-date by logging into Editorial Manager at Editorial Manager® and clicking the ‘Update My Information' link at the top of the page. For questions related to billing, please contact billing support.

If your institution or institutions have a press office, please notify them about your upcoming paper to help maximize its impact. If they’ll be preparing press materials, please inform our press team as soon as possible -- no later than 48 hours after receiving the formal acceptance. Your manuscript will remain under strict press embargo until 2 pm Eastern Time on the date of publication. For more information, please contact onepress@plos.org.

Kind regards,

Chun-Hua Wang

Academic Editor

PLOS One

# Editor report: Acceptance letter

**DOI:** 10.1371/journal.pone.0354840.r004

**Authors:** Chun-Hua Wang

PONE-D-26-18871R1

PLOS One

Dear Dr. SERVIOLE,

I'm pleased to inform you that your manuscript has been deemed suitable for publication in PLOS One. Congratulations! Your manuscript is now being handed over to our production team.

At this stage, our production department will prepare your paper for publication. This includes ensuring the following:

* All references, tables, and figures are properly cited

* All relevant supporting information is included in the manuscript submission,

* There are no issues that prevent the paper from being properly typeset

You will receive further instructions from the production team, including instructions on how to review your proof when it is ready. Please keep in mind that we are working through a large volume of accepted articles, so please give us a few days to review your paper and let you know the next and final steps.

Lastly, if your institution or institutions have a press office, please let them know about your upcoming paper now to help maximize its impact. If they'll be preparing press materials, please inform our press team within the next 48 hours. Your manuscript will remain under strict press embargo until 2 pm Eastern Time on the date of publication. For more information, please contact onepress@plos.org.

You will receive an invoice from PLOS for your publication fee after your manuscript has reached the completed accept phase. If you receive an email requesting payment before acceptance or for any other service, this may be a phishing scheme. Learn how to identify phishing emails and protect your accounts at https://explore.plos.org/phishing.

If we can help with anything else, please email us at customercare@plos.org.

Thank you for submitting your work to PLOS ONE and supporting open access.

Kind regards,

PLOS ONE Editorial Office Staff

on behalf of

Dr. Chun-Hua Wang

Academic Editor

PLOS One
